# Single‐Cell RNA‐Seq Reveals Aging‐Related Impairment of Microglial Efferocytosis Contributing to Apoptotic Cells Accumulation After Retinal Injury

**DOI:** 10.1111/acel.70097

**Published:** 2025-05-15

**Authors:** Pan Liu, Qi Wang, Shuimiao Wang, Ying Liu, Qiqi Chen, Wanyun Qin, Xinna Liu, Xinqi Ye, Yexuan Jiao, Huiping Yuan, Zhengbo Shao

**Affiliations:** ^1^ Department of Ophthalmology The Second Affiliated Hospital of Harbin Medical University Harbin China; ^2^ Future Medical Laboratory The Second Affiliated Hospital of Harbin Medical University Harbin China

**Keywords:** aging, apoptosis, efferocytosis, microglia, retina, single‐cell RNA sequencing

## Abstract

Aging is associated with increased retinal cell apoptosis, which contributes to decreases in retinal function. Apoptotic retinal cell clearance relies on microglial efferocytosis, but the impact of aging on this process has not been fully elucidated. In this study, we aimed to shed light on this by using single‐cell RNA sequencing (sc‐RNA‐seq) to compare young and aged mouse retinal transcriptional profiles, in which 74,412 retinal cells from young and aged mice were classified into 10 transcriptionally distinct retinal cell types, and differentially expressed genes between young versus aged retinas were mainly associated with cellular senescence and apoptosis. Furthermore, ligand–receptor interactions (e.g., AXL‐GAS6, MERTK‐GAS6) between microglia and other retinal cells were strengthened in aged, compared to young retinas. Additionally, among microglia, Subcluster 4 was found under partial clustering to be associated with efferocytosis, of which aged microglia had downregulated efferocytosis‐associated genes. The impact of aging on microglial efferocytosis was further verified in vitro by doxorubicin (DOX)‐induced senescent BV2 microglia, and in vivo by a retinal ischemia/reperfusion (I/R) injury mouse model. In vitro, DOX‐treated BV2 microglia had significantly lowered efferocytosis, as well as efferocytosis‐related MerTK and Axl protein expression; this was also present in vivo in aged retinas post‐I/R injury, with increased co‐localization of ionized calcium‐binding adapter molecule 1^+^ microglia with apoptotic retinal cells, along with reduced efferocytosis‐related protein expression. Overall, microglial efferocytosis of apoptotic cells decreased with aging, suggesting that modulating this process could serve as a possible therapeutic target for age‐related retinal diseases.

## Graphical abstract

Microglia engage with apoptotic cells via cell‐surface receptors, such as proto‐oncogene tyrosine‐protein kinase MER (MerTK) and Axl; these receptors interact with the bridging molecule growth arrest‐specific 6 (Gas6) on apoptotic cells. Young microglia demonstrate efficient efferocytosis of apoptotic cells, but this process is impaired in aged microglia, likely due to lowered expression levels of MerTK and Axl. Consequently, aged retinas, compared to young, have increased accumulation of apoptotic debris, eventually leading to increased inflammation and declines in visual function.

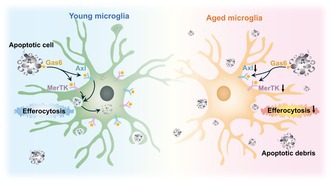

**Abbreviations**
BSA: bovine serum albumin
CD: cluster of differentiation
CDFA: carboxyfluorescein diacetate
CNS: Central nervous system
DAPI: 4′,6‐diamidino‐2‐phenylindole
DEG: differentially expressed gene
DMEM: Dulbecco's modified eagle medium
DOX: doxorubicin
FBS: fetal bovine serum
FITC: fluorescein isothiocyanate
Gas6: growth arrest‐specific 6
GO: Gene Ontology
GSEA: Gene Set Enrichment Analysis
HUVEC: human umbilical cord vein endothelial cell
I/R: ischemia/reperfusion
Iba‐1: ionized calcium‐binding adapter molecule 1
IKK: IκB kinase
IL: interleukin
KEGG: Kyoto Encyclopedia of Genes and Genomes
MerTK: proto‐oncogene tyrosine‐protein kinase MER
NF: nuclear factor
PFA: para‐formaldehyde
PI: propidium iodide
RGC: retinal ganglion cell
SA‐β‐gal: senescence‐associated beta‐galactosidase
sc‐RNA‐seq: single‐cell RNA sequencing
SEM: standard error of the mean
SIRB1: signal regulatory protein β‐1
TNF: tumor necrosis factor
TNFRSF1A: TNF receptor superfamily member 1A
TRITC: rhodamine
TUNEL: terminal deoxynucleotidyl transferase dUTP nick end labeling
UMAP: uniform manifold approximation and projection
VEC: vascular endothelial cell

## 1 Introduction

The retina is a core component of the visual system; it is composed of an intricate network of neurons, which converts light into electrical signals that are conveyed to the visual cortex to generate visual information (Lukowski et al. 2019). Retinal aging, though, results in genomic instability, increased misfolded protein production, mitochondrial dysfunction, and neurotoxic substance accumulation (López‐Otín et al. 2023; Sturgis et al. 2024), all of which result in lowered cellular damage resistance and increased apoptosis (Huang et al. 2022; Zhang et al. 2024). These apoptotic cells, if not removed in a timely manner, contribute to inflammatory responses and produce damage‐associated molecular patterns, subsequently increasing susceptibility to retinal diseases associated with irreversible visual impairment (Coleman‐Belin et al. 2023; Liu et al. 2024).

Previous studies have shown that activated phagocytes play an important role in efferocytosis (Wculek et al. 2022), a process of phagocytic engulfment of apoptotic cells and cell debris (Mehrotra and Ravichandran 2022). Efficient efferocytosis is crucial for timely apoptotic cell removal, along with promoting inflammation resolution, preventing secondary necrosis, and improving microenvironments to favor cell survival. In the central nervous system (CNS), the primary resident immune cells participating in efferocytosis are microglia (Romero‐Molina et al. 2022); in particular, within the retina, they eliminate apoptotic cells and harmful substances generated in damaged tissues to maintain their homeostasis (Shahror et al. 2024). By contrast, defective apoptotic cell efferocytosis by microglia results in inflammatory responses, subsequently accelerating neurodegenerative disease development. One possible factor behind defective efferocytosis may be aging, which has been shown to affect retinal cell function, particularly that of microglia. However, its impact on microglial efferocytosis in the retina still remains incompletely understood.

Traditional microarray and bulk RNA‐sequencing (RNA‐seq) have been used to measure retinal tissue gene expression levels in terms of an average across the entire tissue sample. One limitation of these approaches, though, is that they may conceal the actual heterogeneity in gene expression patterns that are present among a group of cells (Tan et al. 2023). However, this heterogeneity has been recently revealed by single‐cell RNA sequencing (sc‐RNA‐seq), which is able to overcome the limitations of previous technologies. Furthermore, sc‐RNA‐seq findings are independent of classical surface markers for identifying cell subsets. As a result, sc‐RNA‐seq is able to highlight cellular heterogeneity and cell state transitions in unprecedented detail (Sanin et al. 2022), thereby serving as a potentially powerful tool for ophthalmic research, particularly for documenting transcriptional heterogeneity in aged retinas. In this study, we used sc‐RNA‐seq to identify aging‐associated transcriptional changes on microglial efferocytosis in mouse retinas, in which we analyzed different cell types between young and aged retinas, and identified differentially expressed genes (DEGs) associated with apoptosis and efferocytosis. Moreover, we successfully identified an efferocytosis‐associated microglial subcluster, where compared to young, aged microglia had increased intercellular interactions with other retinal cells but decreased efferocytosis; this was verified in both in vivo and in vitro experiments. Therefore, our findings provide important insights into microglial senescence‐associated alterations and highlight a potential novel avenue for treating age‐related retinal diseases.

## 2 Materials and Methods

### 2.1 Acquisition of Mouse Retinas and Preparation of Single‐Cell Suspensions

All animal procedures were approved by the Ethics Committee of the Second Affiliated Hospital of Harbin Medical University (REB#: SYDW2024‐085) and were in accordance with the Guide for the Care and Use of Laboratory Animals (NIH, 8th Edition, 2011). Young (2–3 months) C57BL/6 mice were purchased from the Animal Experiment Center at the Second Affiliated Hospital of Harbin Medical University, and maintained in a specific pathogen‐free environment on a 12‐h light/dark cycle, with food and water available *ad libitum*. Aged mice were obtained by acclimatizing some of those young mice until they were 16–18 months old. Retinas were dissected from the eyeballs of both young and aged mice (3 each), after they were anesthetized, sacrificed, and perfused with ice‐cold saline through the heart.

To prepare single‐cell suspensions, retinas were placed into sterile RNase‐free culture dishes containing Ca^2+^‐ and Mg^2+^‐free 1× PBS, minced into 0.5‐mm^2^ pieces, and washed with PBS. The minced tissues were placed into dissociation solution containing 0.35% collagenase IV, 2 mg/mL papain, and 120 Units/mL DNase I for 20 min in a 37°C water bath with shaking at 100 rpm. The resulting digested tissues were neutralized with PBS containing 10% fetal bovine serum (FBS, v/v) and pipetted 5–10 times with a Pasteur pipette to further dissociate the cells. The cell suspensions obtained were filtered through 70 to 30 μm stacked cell strainers, followed by centrifugation at 300 × g for 5 min at 4°C, and cell pellets resuspended in 100 μL PBS containing 0.04% bovine serum albumin (BSA). One mL of red blood cell lysis buffer (MACS 130‐094‐183, 10×) was added, and the mixture was incubated for 10 min on ice to lyse any remaining red blood cells. Afterwards, single‐cell suspensions were centrifuged at 300 × g for 5 min at room temperature, and dead cells were removed using the Dead Cell Removal Kit (MACS 130‐090‐101, Miltenyi Biotec, Germany), following the manufacturer's instructions. Suspensions were washed three times with PBS containing 0.04% BSA at 300 × g for 3 min at 4°C, and the pellets were resuspended in 50 μL of PBS containing 0.04% BSA. Overall cell viability was confirmed by trypan blue exclusion > 85%, and concentration adjusted to 700–1200 cells/μL.

### 2.2 Building the Chromium 10× Genomics Library and sc‐RNA‐Seq for Mouse Retinal Cells

Single‐cell suspensions were loaded onto the 10× Genomics Chromium Single‐Cell 3′ kit (V3) to capture single cells, following the manufacturer's instructions. The resulting cDNA was amplified, and library construction was performed in accordance with standard protocols. Briefly, single cells with specific 10× Barcode Gel Beads and unique molecular identifiers were partitioned into Gel Bead‐in‐Emulsions within the GemCode instrument and lysed. RNA from those cells was barcoded, reverse‐transcribed, amplified, and sheared, followed by the attachment of 5′ adaptor sequences and sample indexing, to form sequencing libraries, which were sequenced using an Illumina NovaSeq 6000 sequencing system (paired‐end multiplex, 150 bp; LC‐Bio Technology Co. Ltd., China), at a minimum depth of 20,000 reads per cell.

### 2.3 PreProcessing and Clustering Analysis of sc‐RNA‐Seq Data

Preprocessing and quality control analyses of sc‐RNA‐seq data were performed with the 10× Cell Ranger package (v1.2.0; 10× Genomics), in which low‐quality cells with > 25% reads mapped to the mitochondria, or number of genes expressed per cell < 500, were removed. Doublets were also removed with the Doublet Finder R package. After filtration, 74,412 cells were obtained for subsequent analyses. Sequencing data were analyzed with the Cell Ranger software (https://support.10xgenomics.com/single‐cell/software/overview/welcome, accessed 20 August 2022), which uses the STAR aligner (https://github.com/alexdobin/STAR, accessed 20 August 2022) to conduct splice‐aware alignment of RNA‐seq reads to the genome. All these processes yielded gene expression data for individual cells, which were normalized by converting with a scale factor (default setting 10,000), and log‐transformed using the Seurat embedded function (v4.1.0). Correlation analyses were performed using the RunPCA function of the Seurat package.

The top 2000 highly variable genes were extracted from each sample using the “FindVariableFeatures” function, and analyzed by principal component analysis, while main cell clusters were identified using the standard Seurat package, with a 0.8 resolution. To identify subpopulation changes for all cell types within each sample, cell clusters with 0.8 resolution were chosen for sub‐clustering analysis of target cells, and 2‐dimensional visualization was achieved using the “RunUMAP” functions of the Seurat package (dimension parameters 1:30).

### 2.4 Marker Gene and Cell‐Type Annotations

Marker genes for each cell cluster, relative to other cell populations, were first screened using the results of the Seurat FindMarkers algorithm (test. use = bimod). These genes were identified, based on their known cell biology roles, as DEGs for each cell cluster. Distance relationships between cells, within the reduced dimensional uniform manifold approximation and projection (UMAP) map, were also measured.

DEGs were identified from each cell cluster by the “FindMarkers” function in the Seurat package, and statistically significant changes in gene expression between different groups were found using the Wilcoxon rank‐sum test with Bonferroni correction. For multiple hypothesis correction, the Benjamini–Hochberg method was used, and q‐values reflected the false discovery rate. DEGs were determined using the following criteria: (1) Wilcoxon rank‐sum test *q*‐value ≤ 0.01, (2) |log_2_FC| ≥ 0.26, and (3) gene detected in > 10% of cells within a specific cluster.

For cell‐type annotation, the main clusters were manually annotated, based on canonical marker genes and their known functions, as described in previously published reports (Heng et al. 2019; Shekhar and Sanes 2021; Yi et al. 2020; Li et al. 2022). For ambiguous cell subclusters, their annotations were temporarily substituted with numbers. Heatmaps, as well as dot and violin plots for cell‐specific markers, were generated using DoHeatmap, DotPlot, and Vlnplot functions, respectively, in Seurat.

### 2.5 Pathway Enrichment Analyses

Gene Ontology (GO) and Kyoto Encyclopedia of Genes and Genomes (KEGG) enrichment analyses were performed using the clusterProfiler (v3.14.3) R package and hypergeometric distribution. Significantly enriched GO terms and KEGG pathways were identified based on *p*‐adj value < 0.05 and visualized with dot plots. Gene Set Enrichment Analysis (GSEA) was also conducted to determine whether a set of genes exhibited statistically significant differences between young and aged retinas using the OmicStudio platform (https://www.omicstudio.cn).

### 2.6 Partial Cell Clustering

Partial cell clustering analysis was performed by screening cells from previous clustering results. The following parameters were used for clustering and dimensionality reduction analyses: 20 was the number of dimensions used for cell clustering, while for the dimensionality reduction algorithm, the UMAP resolution setting was 0.8. Harmony was used to remove batch effects.

For different clusters designated as FALSE (possible false discoveries) under differential analysis, the following parameters were used: *p* < 0.01 for the bimodal differential screening condition, as well as |log_2_FC| ≥ 0.26. Based on subgrouping classification results, t‐distributed stochastic neighbor embedding and UMAP nonlinear dimensionality reduction methods were then used to visualize the results of Spots subgrouping classification.

### 2.7 Cell–Cell Communication Analysis

Cell–cell communication between various retinal cell types was evaluated using CellPhoneDB (www.cellphonedb.org), based on well‐recognized ligand–receptor pairs from sc‐RNA‐seq data. Ligand–receptor interaction between two cell types were derived, based on a ligand being expressed in one cell type and a receptor in another cell type. The percentage of cells expressing the gene, as well as the average gene expression for each cell type, were calculated. Ligands and receptors expressed by > 10% cells for each cell type were included for the communication analysis. To construct a null distribution for average ligand and receptor expression levels among interacting clusters, cluster labels for all cells were randomly permuted. *p* values, reflecting the likelihood of cell‐type specificity for a given receptor–ligand pair, were calculated based on the proportion of permuted means equal to or higher than the observed mean.

To infer, visualize, and analyze cell–cell communication between different retinal cell types, CellChat (https://github.com/sqjin/CellChat) was used, in which the input consisted of gene expression data from cells, and the probability of intercellular communication was modeled using the law of mass action. The permutation test was then applied to identify significant interactions between two cell types, while key signals and communication patterns were identified by non‐negative matrix factorization, an unsupervised learning algorithm.

### 2.8 Cell Culture

Murine microglia (BV2; AW‐CNM081, Abiowell, China) were cultured in RPMI 1640 (Meilunbio, China), rat retinal cells (R28; EUR201, Kerafast, UK) in low‐glucose Dulbecco's modified eagle medium (DMEM; Cytiva, USA), murine retinal photoreceptor cell line (661W; VCM00070, ViCell, China) in high‐glucose basic DMEM (GIBCO, UK), human umbilical cord vein endothelial cells (HUVECs; HTX1922, Otwo Biotech, China) in DMEM/nutrient mixture F‐12 (GIBCO, UK), and rat retinal Müller cells (rMC‐1; HTX2216, Otwo Biotech, China) in high‐glucose DMEM. Culture mediums for all cell types were supplemented with 10% FBS and 1% penicillin/streptomycin, and were cultured at 37°C in 5% CO_2_.

### 2.9 Inducing BV2 Cell Senescence by Doxorubicin (DOX)

BV2 cells were treated with DOX to induce cell senescence (Wei et al. 2023), in which, upon reaching ~80% confluence, they were exposed to complete medium, supplemented with 200 nM DOX, for 24 h. Cells were then washed with PBS twice, followed by culturing for another 8 h in fresh complete medium. To confirm successful cell senescence induction, senescence‐associated beta‐galactosidase (SA‐β‐gal) staining and Western blot were conducted.

For SA‐β‐gal staining, BV2 cells were plated in 24‐well plates, washed twice with PBS, and fixed with β‐galactosidase staining solution for 15 min at room temperature. Afterwards, cells were washed twice with PBS, incubated with staining solution overnight at 37°C, and imaged using an inverted microscope.

### 2.10 Apoptosis Induction in Retinal Cells and Hoechst/Propidium Iodide (PI) Staining

To induce apoptosis, retinal cells (R28, 661 W, HUVEC, or rMC‐1) were incubated with culture medium containing 50 mM H_2_O_2_ for 10 min, then collected and washed with PBS. Afterwards, Hoechst/PI staining was carried out, in which retinal cells were seeded in a 96‐well plate and incubated with culture medium containing 50 mM H_2_O_2_ for 5 min at room temperature. Cells were incubated with PI (C2015M, Beyotime) at 37°C for 30 min, then washed twice and counterstained with Hoechst 33342 (C1027, Beyotime, China) for 5 min, followed by being observed under a fluorescence microscope (Leica, Germany).

### 2.11 In Vitro Efferocytosis Assay

BV2 cells were treated with or without DOX, as described previously, and washed twice with PBS. Cells were labeled with Dil‐red, washed to remove excess dye, followed by culturing for 30 min in fresh complete medium. Retinal cells (R28, 661 W, HUVEC, or rMC‐1) were treated with H_2_O_2_ to induce apoptosis, labeled with the Vybrant CFDA Cell Tracer Kit (Invitrogen, Carlsbad, CA) for 30 min at room temperature, then washed with PBS twice, each time for 5 min, and resuspended in complete media. Afterwards, BV2 cells were incubated with retinal cells at a 1:1 ratio overnight at 37°C. Non‐ingested retinal cells were removed by PBS washing, and images were captured under a fluorescence microscope (ThermoFisher, USA), in which microglia that engulfed apoptotic cells were double‐positive for Dil/carboxyfluorescein diacetate (CFDA). Efferocytosis was calculated by counting the percentage of Dil^+^CFDA^+^ out of Dil^+^ cells.

### 2.12 Establishing the Retinal Ischemia/Reperfusion (I/R) Injury Mouse Model

The procedure for establishing retinal I/R injury mouse model is described as follows: In brief, both young and old mice were anesthetized with tribromoethanol, and a 32G needle, connected to a saline bottle, was inserted into the anterior chamber of mice eyes. The bottle was raised to 1.5 m, leading to hydrostatic pressure being elevated to 110 mmHg. Mice eyes were instilled with saline for 1 h, followed by removal of the needles to enable retinal vasculature reperfusion.

### 2.13 In Situ Efferocytosis Assay

For in situ efferocytosis, fixed and permeabilized retinal tissue sections were blocked for 30 min, incubated overnight at 4°C with the anti‐ionized calcium‐binding adapter molecule 1 antibody (Iba‐1, 1:100; ab283319, Abcam, USA), followed by fluorescein isothiocyanate (FITC)‐conjugated goat anti‐mouse secondary antibody (1:200; ZF‐0312) for 1 h at room temperature. Afterwards, terminal deoxynucleotidyl transferase dUTP nick end labeling (TUNEL; Roche, Switzerland) staining was conducted, following the manufacturer's instructions, and nuclei counterstained with 4′,6‐diamidino‐2‐phenylindole (DAPI; Beyotime). Efferocytosis index was measured as the percentage of TUNEL^+^Iba‐1^+^ out of Iba‐1^+^ cells.

### 2.14 Immunofluorescence Staining

Eyeballs from both young and old mice, after being subjected to limbal paracentesis with a sterile 32 G syringe, were obtained, fixed overnight in 4% para‐formaldehyde (PFA) at 4°C, and cryo‐protected with increasing percentages of sucrose at 4°C, starting at 10% for 2 h, 20% for 2 h, and 30% for 30 min. The eyeballs were then embedded in OCT compound (Sakura Finetek, Japan), and sectioned through the optic disc of the eye to obtain 5‐μm tissue sections, which were fixed with 4% PFA for 20 min, permeabilized with 0.1% Triton X‐100 in PBS for 10 min, blocked with 10% goat serum in PBS for 2 h, and incubated overnight at 4°C with the following primary antibodies: Iba‐1 (1:100), growth arrest‐specific 6 (Gas6, 1:50; 13,795‐1‐AP, Proteintech, USA), proto‐oncogene tyrosine‐protein kinase MER (MerTK, 1:10; ab300136, Abcam), and Axl (1:10; bs‐5180R, Bioss, USA). Afterwards, sections were incubated with FITC‐conjugated goat anti‐mouse (1:200; ZF‐0312, ZSGB‐BIO, China) or rhodamine (TRITC)‐conjugated goat anti‐rabbit secondary antibodies (1:200; ZF‐0316) for 1 h at room temperature. Nuclei were stained by DAPI at room temperature for 3 min, and cell apoptosis was detected by TUNEL. The preparation procedure for retinal flatmounts is outlined below: Retinas isolated from mouse eyeballs were fixed with 4% PFA for 1 h, permeabilized overnight with 0.5% Triton X‐100 in PBS at 4°C, and blocked with 10% goat serum in PBS for 1 h. Retinas were then incubated overnight with Iba‐1 (1:200) at 4°C, then with TRITC‐conjugated goat anti‐rabbit secondary antibody for 1 h at room temperature. Slides were mounted using Dako anti‐fade fluorescence mounting medium (S3023, Denmark). Images were obtained under a fluorescence microscope (ThermoFisher, USA) or confocal laser scanning microscopy (Carl Zeiss, Germany), and fluorescence intensity was quantified by ImageJ.

### 2.15 Western Blot

Retinas and BV2 cells were lysed by RIPA lysis buffer (Beyotime) for protein extraction, and proteins were quantified using the BCA Protein Assay Kit (Solarbio, China). Twenty μg of total protein was separated by SDS‐PAGE gel electrophoresis (Epizyme, China), transferred to polyvinylidene difluoride membranes (Millipore, Germany), and incubated with the following primary antibodies: CX3CL1 (1:1000; 60339‐1‐Ig, Proteintech), CX3CR1 (1:1000; ab308613, Abcam), Gas6 (1:50), Axl (1:100), MerTK (1:100), Bax (1:100; R22708, Zen‐Bioscience, China), Bcl‐2 (1:100; R23309), P53 (1:50; 10442‐1‐AP, Proteintech), P16 (1:100; R23896, ZEN BIO), and β‐actin (1:8000; 66009‐1‐Ig, Proteintech). Membranes were then incubated with horseradish peroxidase‐conjugated secondary antibody. Protein expression was detected using ECL detection reagent, and bands were visualized using an imaging system (ChemiDoc, USA). Protein expression levels were quantified by ImageJ, normalized to the housekeeping gene β‐actin.

### 2.16 Statistical Analysis

Statistical analyses were conducted using GraphPad Prism (version 9.5.0). Data was presented as mean ± standard error of the mean (SEM). Comparisons between two groups were carried out using unpaired two‐tailed Student's *t*‐test. The interaction strengths ligand–receptor pairs between microglia and different cell types were compared using a paired Student's *t*‐test. *p* < 0.05 was considered statistically significant, unless otherwise stated.

## 3 Results

### 3.1 sc‐RNA‐Seq Profiling of Young and Aged Mouse Retinas to Identify Different Cell Types

**FIGURE 1:**
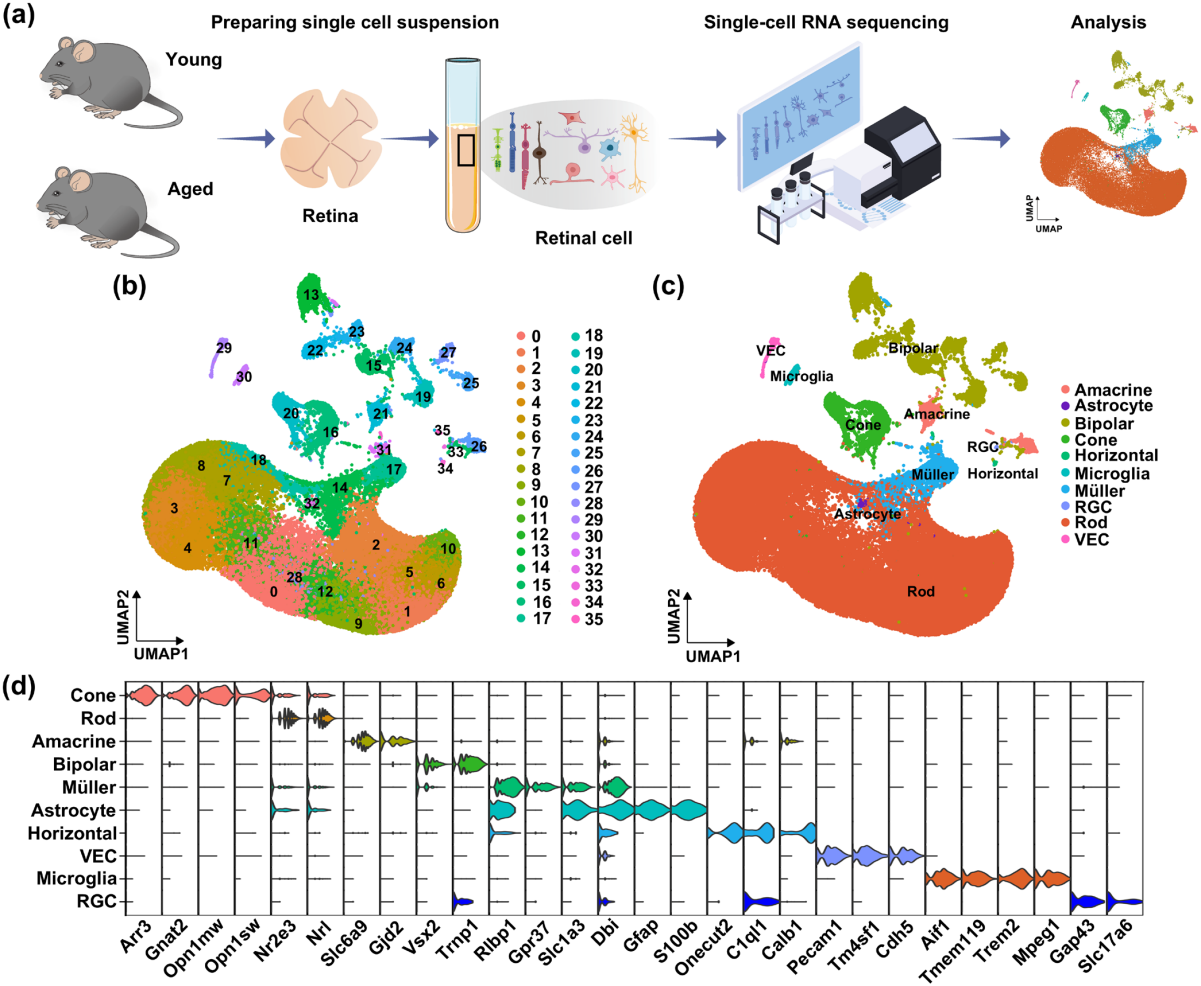
Identification of different retinal cell types using single‐cell RNA sequencing (sc‐RNA‐seq). (a) Flowchart depicting the sc‐RNA‐seq workflow for 3 young and 3 aged mouse retinas. (b) Uniform manifold approximation and projection (UMAP) plot of the transcriptional profiles from 74,412 retinal cells from the 3 young and 3 aged retina samples, falling into 35 clusters. (c) Clustering analysis further categorized the retinal cells into 10 different cell types (amacrine, astrocyte, bipolar, cone, horizontal, microglia, Müller, retinal ganglion [RGC], rod, and vascular endothelial [VEC]), based on canonical cell markers. (d) Violin plots of expression levels (logarithmic‐scale) for the canonical cell markers associated with the 10 different retinal cell types. *n* = 3/group.

Figure 1a is a flowchart showing the sc‐RNA‐seq workflow, in which retinal tissues were extracted from 3 young and 3 aged mice, and single‐cell suspensions were prepared from them, which were loaded onto the 10× Genomics platform to generate barcoded sc‐RNA‐seq libraries. All six sequenced samples exceeded the standard quality benchmark of > 20,000 reads/cell, with individual specimens demonstrating Mean Reads/Cell values ranging from 28,768 to 45,885. Additionally, after applying quality control procedures to exclude low‐quality cells and doublets, 74,412 retinal cells were retained for downstream analyses. Unsupervised clustering of these cells via UMAP dimensionality reduction revealed 35 transcriptionally distinct cell clusters (Figure 1b and Table S1). These cells could be further categorized into 10 different retinal cell types, based on previously identified canonical cell markers (Heng et al. 2019; Shekhar and Sanes 2021; Yi et al. 2020; Li et al. 2022) (Figure 1c), including rod (Arr3, Gnat2, Opn1mw, Opn1sw), cone (Nr2e3, Nrl), amacrine (Slc6a9, Gjd2), bipolar (Vsx2, Trnp1), horizontal (Onecut2, C1ql1, Calb1), retinal ganglion cells (RGC; Gap43, Slc17a6), Müller (Rlbp1, Gpr37, Slc1a3, Dbi), astrocyte (Gfap, S100b), microglia (Aif1, Tmem119, Trem2, Mpeg1), and vascular endothelial cells (VEC; Pecam1, Tm4sf1, Cdh5) (Figure 1d). These findings thus demonstrated that sc‐RNA‐seq successfully identified different retinal cell types, based on their transcriptional profiles.

### 3.2 DEG Analysis of Retinal Cell Types Revealed That Aged Cells Had Increased Apoptotic Activity

**FIGURE 2:**
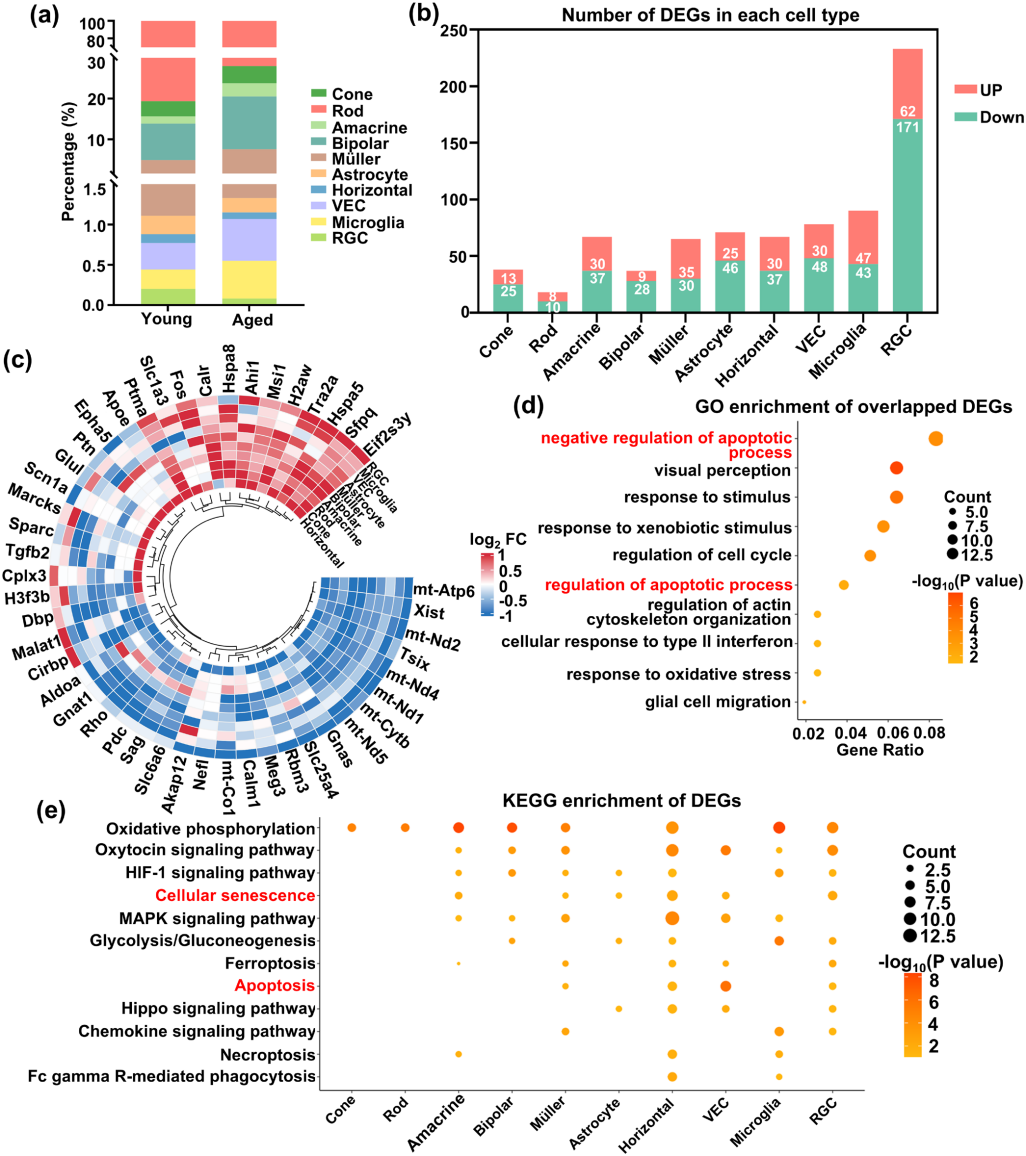
Differentially expressed gene (DEG) analysis between 10 different cell types from young and aged retinas. Stacked bar graphs, depicting (a) percentages of the 10 retinal cell types between young and aged retinas, as well as (b) up‐ and downregulated DEGs for each retinal cell type in aged, compared to young retinas. (c) Heatmap showing overlapping DEGs among the 10 retinal cell types. Bubble plots of (d) Gene Ontology (GO) enrichment analysis of overlapped DEGs, as well as (e) Kyoto Encyclopedia of Genes and Genomes (KEGG) enrichment analysis of DEGs from the 10 retinal cell types, among aged versus young retinas. *n* = 3/group.

To ascertain the effect of aging on retinal cell populations, we compared the proportions of the 10 retinal cell types between young and aged mice. We found that compared to young, aged retina had increased proportions of amacrine, bipolar, VECs, Müller, and microglia, along with decreased RGCs and rod cells (Figure 2a). We then assessed the number of DEGs for each cell type, finding that compared to young, aged retinas had significant changes in gene expression among amacrine, horizontal, Müller, astrocytes, microglia, VECs, and RGCs (Figure 2b). A number of overlapping DEGs among these retinal cell types were also identified (Figure 2c), which were found under GO enrichment analysis to be mainly associated with negative regulation of apoptosis, visual perception, and response to stimulus (Figure 2d). Additionally, DEGs among aged versus young retinal cells were found under KEGG to be enriched for oxidative phosphorylation, oxytocin signaling pathway, HIF‐1 signaling pathway, cellular senescence, ferroptosis, and apoptosis pathways (Figure 2e). Collectively, these observations suggested that aged retinal cells had increased proapoptotic gene expression and activity.

### 3.3 Aged Microglia Exhibited Enhanced Intercellular Communication With Other Retinal Cells and Upregulation of Inflammatory‐Related Signaling Pathways

**FIGURE 3:**
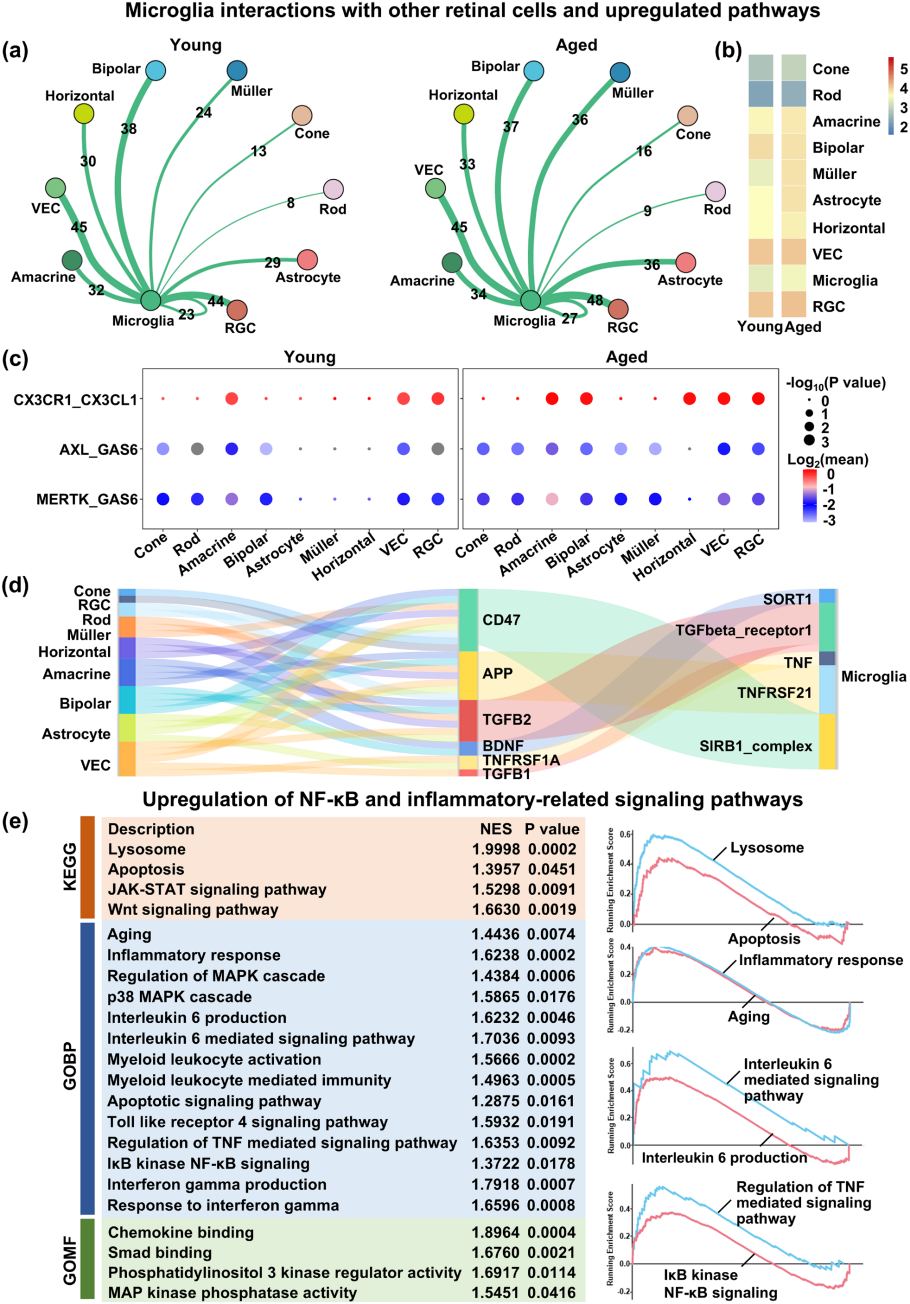
Aged microglia had increased cell–cell communication with other retinal cells and upregulation of inflammatory‐related signaling pathways. (a) Network plots and (b) heatmap depicting the number of interactions between microglia and other retinal cell types in young and aged retinas. (c) Bubble plot showing the strength of interactions between efferocytosis‐related ligands (CX3CL1, GAS6) on other retinal cell types and microglial receptors (CX3CR1, AXL, MERTK) in young and aged retinas. (d) Sankey plot showing interactions between microglia and other retinal cell types in aged retinas. (e) Gene set enrichment analysis (GSEA) between young and aged microglia, depicting significantly enriched terms with their normalized enrichment scores (NES) (left), as well as enrichment plots for selected terms in microglia (right). *n* = 3/group.

Microglia, as the primary executor of apoptotic cell clearance in the CNS, were found to have increased abundance in the aged retina, based on sc‐RNA‐seq analysis and validation experiments (Figure 2a and Figure S1). Consequently, we further investigated aging‐related molecular and functional alterations in microglia. CellPhoneDB was used to interpret intercellular communications between microglia and other retinal cells, and interactions with significant *p* values (*p* < 0.05) were identified; the number of interactions specific to, or shared between, young and aged retinas is shown in Figure S2a. Compared to young, aged microglia had increased numbers of interactions with cone, Müller, astrocyte, and RGCs (Figure 3a,b). We also examined ligand–receptor interaction strengths between microglia and different cell types in both young and aged retina, and observed that aged microglia, compared to young, had stronger interaction strengths with multiple cell types, including cone, rod, amacrine, bipolar, Müller, and VECs (Figure S2b).

Furthermore, several ligand–receptor interactions, such as CX3CR1‐CX3CL1, AXL‐GAS6, and MERTK‐GAS6, were closely related to efferocytosis. Consequently, we explored the interaction strengths of these ligand–receptor interactions between microglia and other retinal cell types, which were calculated based on receptor and ligand expression levels. We found that the interaction strength of CX3CR1‐CX3CL1 increased with aging for microglia–bipolar and microglia–horizontal interactions, while AXL‐GAS6 interaction strength increased for microglia–rod, microglia–amacrine, microglia–astrocyte, microglia–Müller, and microglia‐RGC. As for MERTK‐GAS6, aged microglia had increased interaction strength with almost all retinal cells (Figure 3c). All these findings thus indicated that aged microglia had increased interactions with other retinal cells. Additionally, interactions between signal regulatory protein β‐1 (SIRB1) and cluster of differentiation (CD) 47, as well as tumor necrosis factor (TNF) and TNF receptor superfamily member 1A (TNFRSF1A), have been observed between microglia and other cells in aged retinas (Figure 3d), which have been associated with inflammation and nuclear factor (NF)‐κB activity (Doran et al. 2020). This association was further supported by GSEA, which revealed that aging was associated with upregulation of genes associated with inflammatory responses, IκB kinase (IKK)‐NF‐κB signaling, as well as interleukin (IL)‐6‐ and regulation of TNF‐mediated signaling pathways in microglia (Figure 3e).

### 3.4 Identifying Microglial Heterogeneity and the Subcluster Involved in Efferocytosis

**FIGURE 4:**
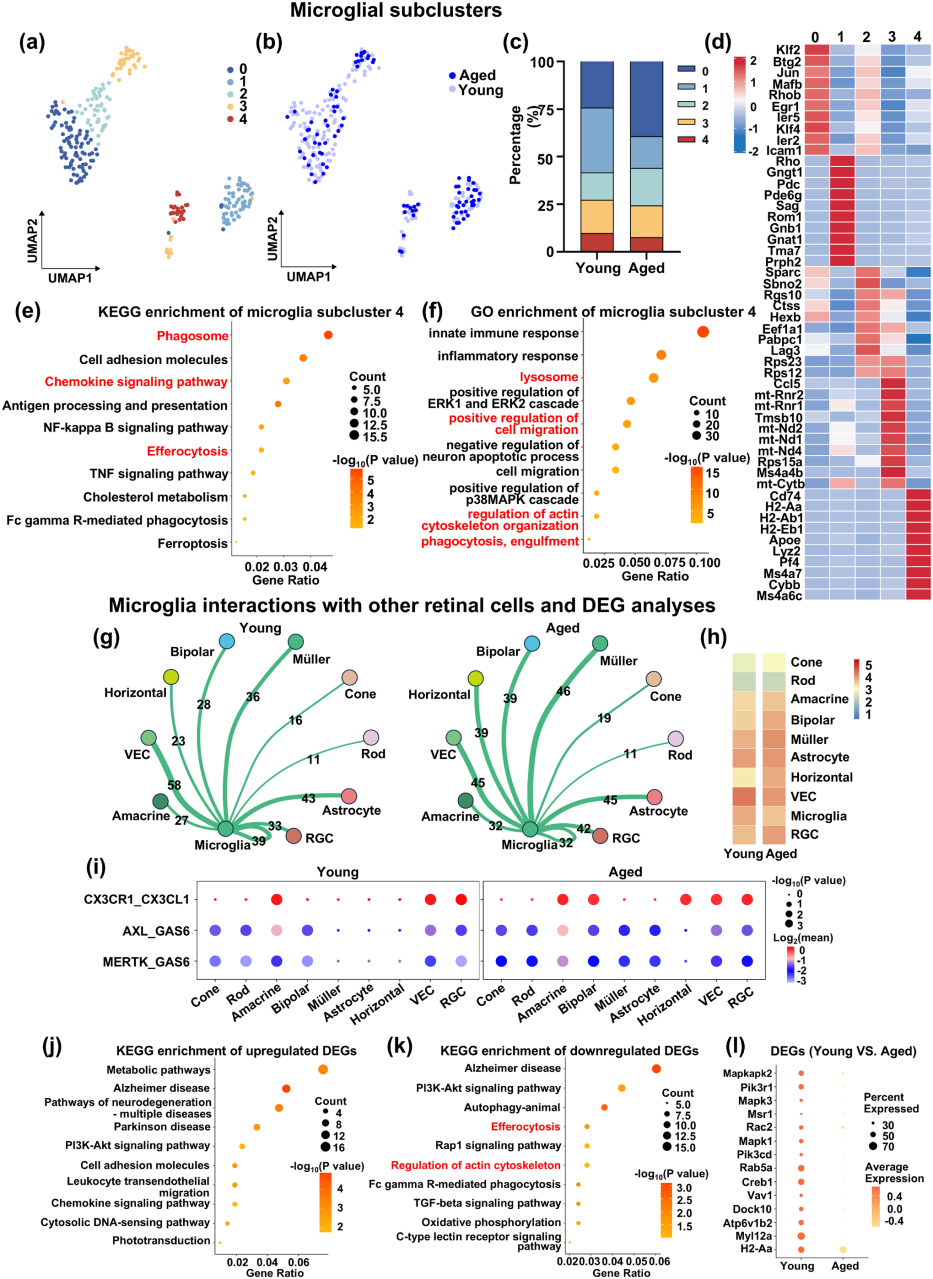
Partial cell clustering analysis revealed that aged microglial Subcluster 4 was associated with efferocytosis; this subcluster also had increased intercellular interactions with other retinal cells and downregulation of efferocytosis‐related DEGs. UMAP plots of 259 young and aged microglia, showing (a) five microglia subclusters (0–4), as well as (b) distinguishing between young and aged microglia. (c) Stacked bar graph showing percentages of the five subclusters between young and aged microglia. (d) Heatmap showing the top 10 marker genes used to identify each of the five microglial subclusters. Bubble plots of (e) KEGG and (f) GO enrichment analyses of DEGs from microglial Subcluster 4. (g) Network plots and (h) heatmap depicting the frequency of communications between Subcluster 4 microglia and other retinal cell types, in young and aged retinas. (i) Bubble plot showing the strength of interactions between efferocytosis‐related ligands on other retinal cell types and Subcluster 4 microglial receptors, in young and aged retinas. Bubble plots of KEGG enrichment analyses for (j) up‐ and (k) downregulated DEGs in aged Subcluster 4 microglia, as well as (l) Bubble plot showing efferocytosis‐related gene expression between young and aged Subcluster 4 microglia. *n* = 3/group.

Based on the findings of increased interactions between aged microglia and other retinal cells, we examined the effect of aging on microglial heterogeneity by conducting partial clustering analysis of microglia, using UMAP. We found a total of 259 young and aged microglia, which were divided into five distinct subclusters, labeled 0–4 (Figure 4a,b). Compared to young, aged microglia had increased proportions of subcluster 0 and 2, as well as decreases in Subcluster 1 and 4 (Figure 4c). Figure 4d shows the top 10 marker genes used to identify each microglial subcluster, in which Subcluster 4 expressed high levels of Cd74, H2‐Aa, H2‐Ab1, H2‐Eb1, Apoe, Lyz2, Pf4, Ms4a7, Cybb, and Ms4a6c.

The functions associated with these microglial subclusters were then identified by KEGG, in which for Subcluster 4, it was enriched for phagosome, chemokine signaling, and efferocytosis (Figure 4e). This was not the case for other subclusters, such as 0 being enriched for MAPK signaling, 1 for neurodegeneration‐multiple diseases, 2 for lysosome, and 3 for neurodegeneration‐multiple diseases and oxidative phosphorylation (Figure S3a–d).

Likewise, Subcluster 4 was found under GO analysis to be enriched for lysosome, positive regulation of cell migration, regulation of actin cytoskeleton organization, as well as phagocytosis, engulfment (Figure 4f). By contrast, for Subclusters 0–3, other cellular processes were enriched, such as innate immune response for Subcluster 0, nervous system development for 1, ribosomal small subunit biogenesis for 2, and rRNA processing for 3 (Figure S3e–h). Therefore, both KEGG and GO revealed that microglia Subcluster 4 was most strongly linked to DEGs and pathways associated with efferocytosis, which was confirmed by the identification of genes involved in efferocytosis being highly expressed in that subcluster (Figure S3i). Consequently, microglial Subcluster 4 may be the primary one responsible for efferocytosis.

### 3.5 Aged Microglial Subcluster 4 Had Increased Intercellular Interactions With Other Retinal Cells and Downregulation of Efferocytosis‐Related DEGs

We examined cell–cell interactions between young or aged microglia from Subcluster 4 with other retinal cell types and found that compared to young, aged Subcluster 4 microglia had more interactions with cone, amacrine, bipolar, Müller, astrocyte, horizontal, and RGCs (Figure 4g,h). Additionally, aged Subcluster 4 had stronger CX3CR1‐CX3CL1 interaction strengths with bipolar and horizontal cells, as well as AXL‐GAS6 and MERTK‐GAS6 with all but horizontal cells versus young microglia (Figure 4i).

Furthermore, KEGG analysis found that aged microglial Subcluster 4, compared to young, had enrichment of upregulated genes for pathways of neurodegeneration‐multiple diseases (Figure 4j), and downregulated genes for efferocytosis, regulation of actin cytoskeleton, Fc gamma R‐mediated phagocytosis, TGF‐beta signaling pathway, and oxidative phosphorylation (Figure 4k). Correspondingly, a large number of genes involved in efferocytosis, such as Msr1, Rac2, Mapk1, Creb1, Vav1, Dock10, Atp6v1b2, My112a, and H2‐Aa, were downregulated in aged microglia Subcluster 4 (Figure 4l). Therefore, aged Subcluster 4 microglia, compared to young, had increased interactions with other retinal cells, along with downregulation of efferocytosis‐related genes. In addition, the upregulated DEGs in aged microglial Subcluster 4, compared to their young counterparts, were found under GO analysis to be enriched for apoptotic process, as well as positive regulation of canonical NF‐κB signal transduction and inflammatory cytokine production, such as TNF, IL‐1β, and IL‐6 (Figure S4a,b). Afterwards, we further analyzed cell–cell communication in young and aged retinal cells with CellChat, in which, compared to young, the TNF signaling pathway was upregulated in aged microglia Subcluster 4, while insulin‐like growth factor (IGF)‐1 signaling pathway was downregulated in aged RGCs (Figure S4c,d).

### 3.6 DOX‐Induced Senescent BV2 Microglia Had Lowered Efferocytosis of Apoptotic Cells

We then examined whether the sc‐RNA‐seq findings of aging being associated with the downregulation of microglial efferocytosis were also present in an in vitro model of senescent BV2 murine microglia. Based on previous reports of DOX being able to induce microglial senescence (Wei et al. 2023), we treated BV2 cells with DOX and found that DOX‐treated BV2 (BV2 + DOX), compared to untreated control (BV2), had increased SA‐β‐gal^+^ (Figure S5a,b), p53 and p16 expression (Figure S5c–e), indicating successful induction of BV2 senescence.

**FIGURE 5:**
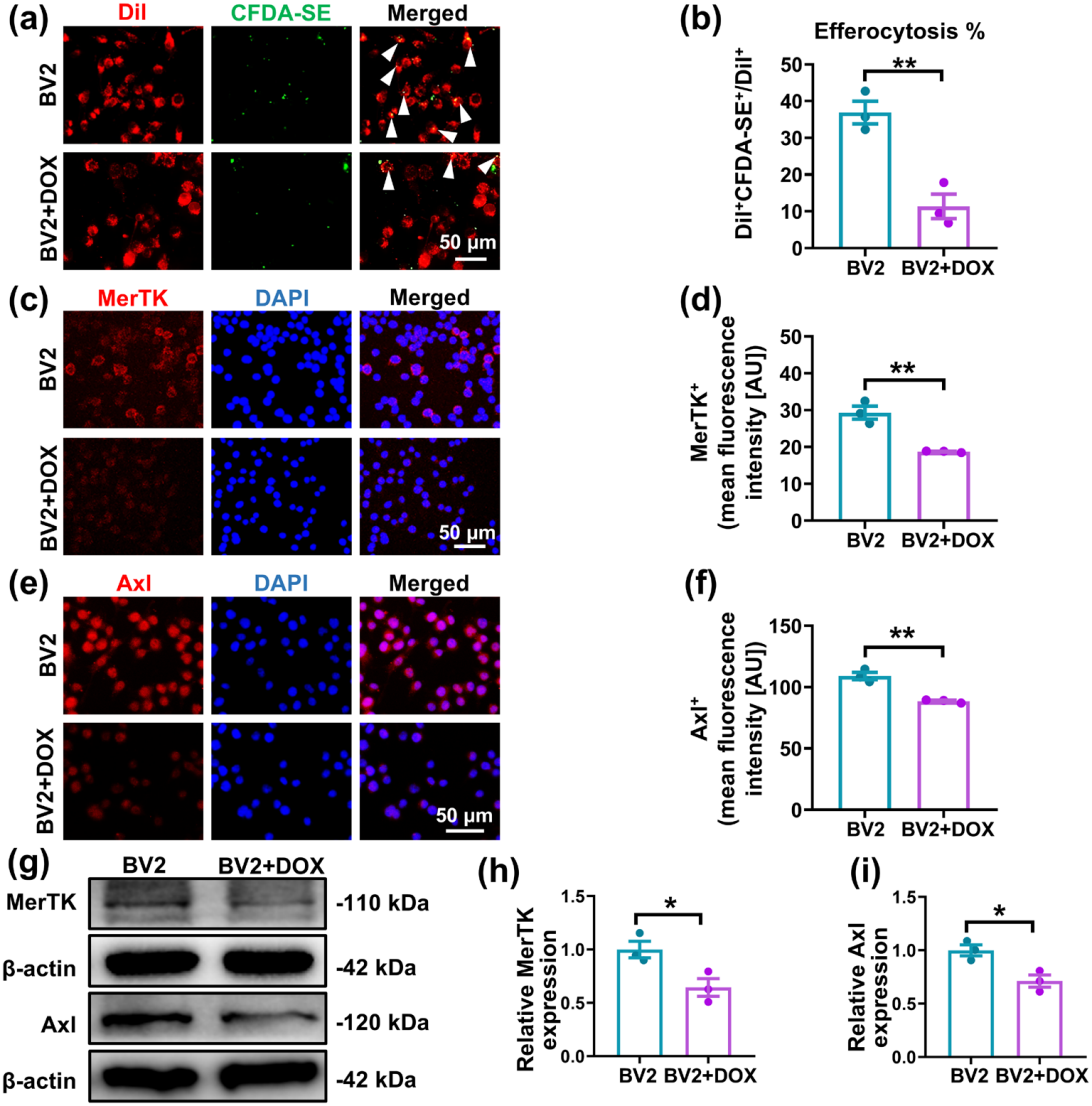
Doxorubicin (DOX)‐treated senescent BV2 murine microglia had lowered efferocytosis of apoptotic retinal cells in an in vitro model. (a) Representative immunofluorescence staining images and (b) quantification of Dil^+^carboxyfluorescein diacetate (CFDA)^+^ cells, representing successful efferocytosis of apoptotic (induced by H_2_O_2_) CFDA^+^ R28 rat retinal cells by Dil^+^ BV2 microglia, as a percentage of total Dil^+^, between untreated control (BV2) and DOX‐treated senescent BV2 (BV2 + DOX) groups. (c) Representative immunofluorescence images and (d) quantification of mean fluorescence intensity for proto‐oncogene tyrosine‐protein kinase MER (MerTK)^+^ cells between the two groups. Nuclei were stained with 4′,6‐diamidino‐2‐phenylindole (DAPI). (e) Representative immunofluorescence images and (f) quantification of mean fluorescence intensity for Axl^+^ between the two groups. (g) Representative Western blot image, as well as quantification of (h) MerTK and (i) Axl protein expression levels between the two groups. Protein expression was normalized to β‐Actin. Data are expressed as mean ± standard error of the mean (SEM). *n* = 3/group. *p < 0.05, **p < 0.01.

To measure the extent of efferocytosis, both BV2 and BV2 + DOX cells were labeled with Dil and incubated with CFDA‐labeled apoptotic R28 rat retinal cells. R28 apoptosis was successfully induced by H_2_O_2_ (R28 + H_2_O_2_), as confirmed by the increase in PI^+^ cells versus untreated R28 (Figure S5f,g). The percentage of Dil^+^CFDA^+^, representing successful efferocytosis out of all Dil^+^ cells, was significantly lower among BV2 + DOX compared to BV2, indicating that senescent microglia had impaired efferocytosis (Figure 5a,b). BV2 and BV2 + DOX cells were also cocultured with H_2_O_2_‐treated apoptotic 661 W, HUVEC, and rMC‐1 cells; this yielded similar results to those of BV2 and BV2 + DOX with apoptotic R28, in which BV2 + DOX had lowered efferocytosis of those three apoptotic cell types (Figure S6a–f).

This was further supported by immunofluorescence staining for MerTK and Axl, which are critical receptors for apoptotic cell recognition. The results revealed that BV2 + DOX had significantly lower MerTK^+^ and Axl^+^ fluorescence intensity than BV2 (Figure 5c–f). The same observations were also found from Western blot analysis, where protein expression levels of MerTK and Axl significantly decreased in BV2 + DOX versus BV2 (Figure 5g–i). All these in vitro findings thus demonstrate that aging, in the form of DOX‐induced senescence, impaired efferocytosis, likely by reducing the expression of efferocytosis‐related receptor proteins.

### 3.7 Aging Increased Retinal Cell Apoptosis and Lowered Microglial Efferocytosis Post‐I/R Injury

**FIGURE 6:**
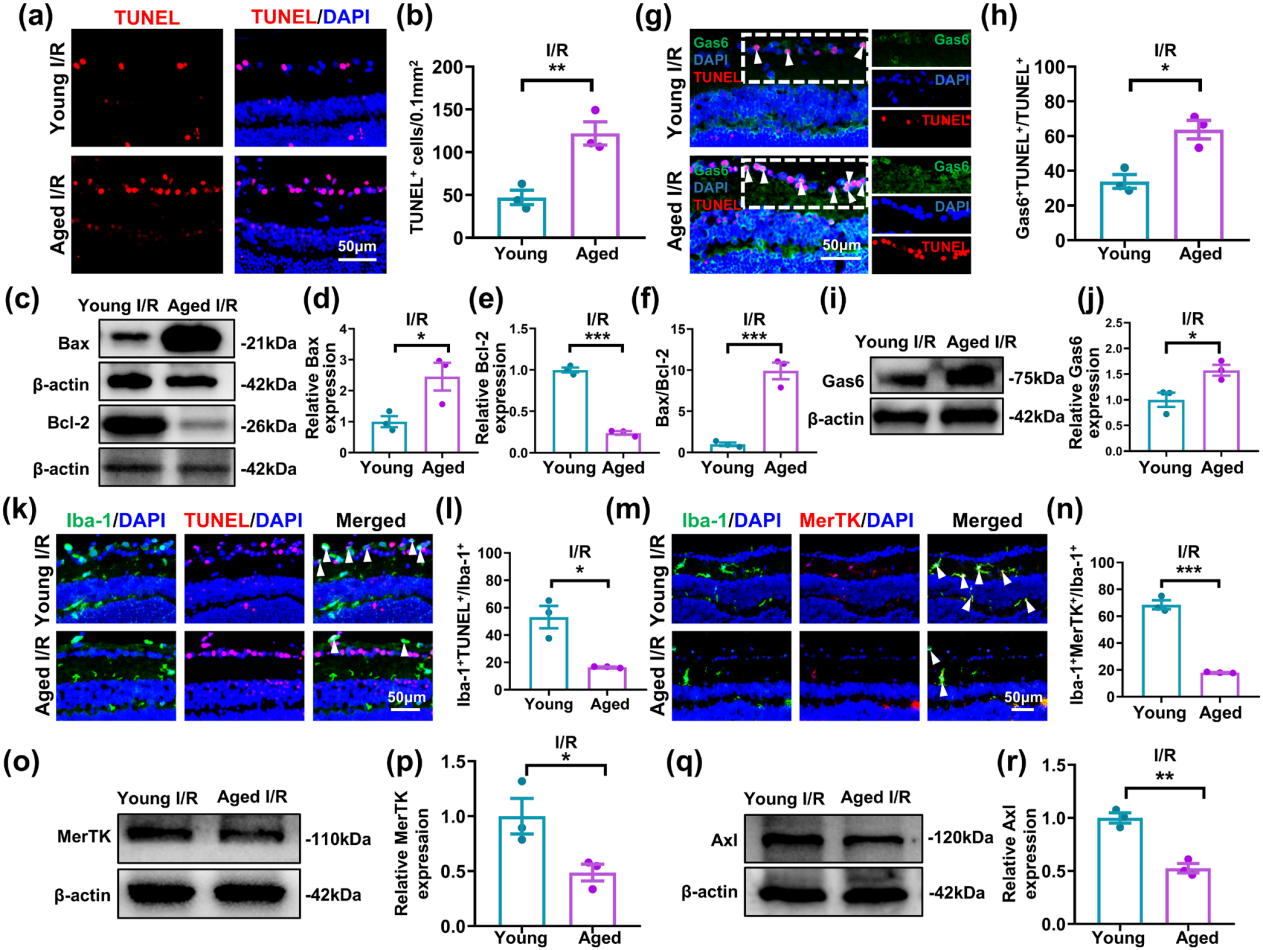
Aged retinas, compared to young, had increased apoptotic cells and lowered microglial efferocytosis post‐ischemia/reperfusion (I/R) injury in an in vivo mouse model. (a) Representative immunofluorescence images and (b) quantification of apoptotic terminal deoxynucleotidyl transferase dUTP nick end labeling (TUNEL)^+^ retinal cells between young (Young I/R) and aged (Aged I/R) retinas post‐I/R injury. Nuclei were stained with DAPI. (c) Representative Western blot images, as well as quantification of (d) pro‐apoptotic Bax, (e) anti‐apoptotic Bcl‐2, and (f) Bax/Bcl‐2 protein levels between the two groups. (g) Representative immunofluorescence images and (h) quantification of growth arrest‐specific 6 (Gas6)^+^TUNEL^+^ within the ganglion cell layer for the two groups. (i) Representative Western blot image and (j) quantification of Gas6 protein expression between the two groups. (k) Representative immunofluorescence images and (l) quantification of ionized calcium‐binding adapter molecule 1 (Iba‐1)^+^ microglia co‐localized with apoptotic TUNEL^+^ retinal cells, out of all Iba‐1^+^, on Day 3 post‐I/R injury, between young and aged retinas. (m) Representative immunofluorescence images and (n) quantification of Iba‐1^+^MerTK^+^, out of all Iba‐1^+^ microglia, between the two groups. (o) Representative Western blot image and (p) quantification of MerTK protein expression between the two groups. (q) Representative Western blot image and (r) quantification of Axl protein expression between the two groups. Protein expression was normalized to β‐actin. Data are expressed as mean ± SEM. *n* = 3/group. *p < 0.05, **p < 0.01, ***p < 0.001.

I/R injury is a pathological process occurring in multiple aging‐associated retinal diseases, including glaucoma, central retinal artery and vein occlusions, and diabetic retinopathy. To determine whether aging also affected microglial efferocytosis in vivo, as suggested from sc‐RNA‐seq and in vitro observations, a retinal I/R injury mouse model was established, in which among aged mouse retinas (Aged I/R), significantly higher TUNEL^+^ cells were present compared to young mice (Young I/R; Figure 6a,b). In accordance with this finding, aged I/R had significantly higher proapoptotic Bax and lower anti‐apoptotic Bcl‐2 protein expression compared to young I/R (Figure 6c–f). Therefore, aged retinas had increased apoptosis compared to young ones post‐I/R. We also examined the expression levels of efferocytosis‐related receptors and ligands between young versus aged retina, in which under normal physiological conditions, no significant differences in CX3CR1 and CX3CL1 expression levels were present between young and aged retinas (Figure S7a–c). This was not the case for MerTK and AXL, though, as their levels were lower in aged retinas than young ones (Figure S7d–f).

Next, we examined the ability of microglia in the retinal I/R injury model to bind to apoptotic cells, via the bridging molecule Gas6, and found that aged I/R, compared to young, had significantly increased numbers of Gas6^+^TUNEL^+^ cells in the ganglion cell layer (Figure 6g,h). This was also supported by Western blot analysis, where Gas6 protein expression was significantly higher in aged versus young I/R (Figure 6i,j). We then evaluated microglial efferocytosis by quantifying the number of Iba‐1^+^ microglia co‐localized with TUNEL^+^ apoptotic retinal cells, in which the proportions of Iba‐1^+^TUNEL^+^ cells was significantly lower in aged retinas (Figure 6k,l and Figure S8a,b). Similarly, post‐I/R, aged retinas, compared to young, also had lowered Iba‐1^+^MerTK^+^ cells (Figure 6m,n and Figure S8c). Furthermore, Western blot analysis showed that just like for in vitro DOX‐treated BV2 microglia, aged I/R had significantly lower MerTK and Axl protein expression versus young (Figure 6o–r). All these results thus demonstrated that after I/R injury, apoptotic cells were increased, while microglial efferocytosis was impaired, in aged retinas.

## 4 Discussion

Aging increases risks for a variety of retinal diseases, but the potential mechanisms involved in contributing to those increased risks require further exploration. In this study, sc‐RNA‐seq was used to identify transcriptional profile changes in aged retinas. We comprehensively documented senescence‐associated gene expression alterations by identifying and analyzing cell‐type composition, intercellular interactions, as well as cell subpopulations. Our major findings were the successful identification of (1) 10 cell types, based on canonical retinal marker genes, (2) cellular senescence/apoptosis‐related DEGs being enriched in amacrine, horizontal, VEC, Müller, and RGCs, (3) aged, compared to young microglia, having increased interactions with other retinal cells, (4) the efferocytosis‐associated microglia subpopulation, based on specific gene expression, and (5) microglial efferocytosis decreasing with retinal aging, which was observed both in vitro, among DOX‐induced senescent BV2, as well as in vivo, in a retinal I/R injury mouse model.

Aging has been documented to lead to retinal structural and functional changes (Liu et al. 2024). In fact, several studies have revealed that retinal cell numbers changed with aging (Coleman‐Belin et al. 2023; Liu et al. 2024). More specifically, our sc‐RNA‐seq data pinpointed that aging mainly affected specific retinal cells, such as microglia and RGC, but minimally impacted on rod, cone, and bipolar cells. Our identification of microglia, the immune cells of the retina, as being particularly affected by aging is supported by previous investigations showing that microglial proportions increase in aged retinas (Liu et al. 2024). As for RGCs, they have been documented to be progressively lost with aging (Ju et al. 2023), thereby serving as one of the major risk factors for glaucoma (Jayaram et al. 2023). Indeed, our study found that RGCs were among the retinal cell types most susceptible to aging, which supports the association between RGCs and glaucoma pathogenesis. Aging also alters the retinal microenvironment, which is accompanied by increased apoptosis (Zhang et al. 2024). Our sc‐RNA‐seq analysis revealed that DEGs between young and aged retinal cells were related to apoptosis and ferroptosis. Yet, even though large numbers of apoptotic cells are routinely generated, they are hardly observable in vivo, due to apoptosis being tightly coupled with highly effective phagocytic removal, in a process termed efferocytosis (Boada‐Romero et al. 2020). In the retina, efferocytosis is performed by resident microglia, in which quick removal of apoptotic retinal cells prevents them from undergoing secondary necrosis, where they release toxic intracellular contents that could induce inflammation (Shahror et al. 2024).

Microglia can be divided into different subtypes, which usually perform different functions, including inflammatory resolution, pro‐inflammatory, immunomodulatory, and phagocytosis (Sanin et al. 2022). Different microglial subclusters, though, cannot be effectively identified by the traditional method based on surface markers (Shi et al. 2022). With the advances of sc‐RNA‐seq, these subclusters can be identified, and overall microglial heterogeneity elaborated, based on gene expression levels. Notably, our sc‐RNA‐seq findings were able to identify a specific microglia subcluster responsible for efferocytosis, based on it being enriched for a large number of efferocytosis‐related genes. The subcluster was also enriched for genes associated with the chemokine signaling pathway, cell migration, regulation of actin cytoskeleton organization, phagosome, and lysosome, all of which are efferocytosis‐associated biological processes (Mehrotra and Ravichandran 2022), thereby further supporting that this microglia subcluster was involved in efferocytosis. We then focused on the effects of aging on that efferocytosis‐associated microglia subcluster and observed that aged microglia exhibited enhanced intercellular interactions with other retinal cells. One possible explanation is that aged retinas had increased apoptosis, in turn necessitating their removal by microglia via efferocytosis. With respect to retinal efferocytosis, microglia first locate apoptotic retinal cells by binding their receptors (e.g., CX3CR1) to ligands (CX3CL1) released by apoptotic cells (Doran et al. 2020). However, aging has had little effect on CX3CR1‐CX3CL1 interactions between microglia and other retinal cells, indicating that the age‐related impact on microglial migration toward apoptotic retinal cells is relatively limited.

In fact, a mixed picture has been found, in which some studies, such as by Huang et al. found that CX3CR1‐CX3CL1 interactions between microglia and retinal photoreceptor cells resulted in microglial activation and increased photoreceptor degeneration in a mouse retinal degeneration model (Huang et al. 2024). Additionally, Subbarayan et al. noted that the soluble isoform of CX3CL1 was associated with neurodegeneration, while the membrane‐bound version was neuroprotective (Subbarayan et al. 2022). These studies, though, were contradicted by Yu et al. who found that the CX3CR1‐CX3CL1 interaction between microglia and RGCs exerted a neuroprotective effect on RGCs in a mouse optic nerve trauma model (Yu et al. 2024). Our findings were in line with Yu et al. in that CX3CR1‐CX3CL1 interactions were also found between microglia and RGCs, but they were among both young and old microglia, indicating that aging did not significantly impact this microglial‐retinal cell interaction. Indeed, no quantitative differences in CX3CL1 or CX3CR1 protein expression were present under Western blot analysis between young and aged retinas. The association between CX3CR1‐CX3CL1 interactions and positive neuronal function was further supported by Pawelec et al. who observed that disrupting these interactions was associated with the exacerbation of neurodegenerative disorders, such as amyotrophic lateral sclerosis (Pawelec et al. 2020), as well as by Roche et al. who observed that administering Norgestrel resulted in increased photoreceptor survival via increasing CX3CR1‐CX3CL1 signaling (Roche et al. 2017). However, future studies are required to clarify this ambiguous picture regarding the possible outcomes of CX3CR1‐CX3CL1 interactions between microglia and other retinal cells, as well as the impact of aging on those outcomes.

Other ligand–receptor interactions, such as MERTK‐GAS6 and AXL‐GAS6, in which microglia engage apoptotic retinal cells through cell‐surface receptors (such as MerTK and Axl) that either directly bind molecules or indirectly bind soluble bridging molecules (Gas6 and Protein S), in turn enabling them to interact with phosphatidylserine on apoptotic cells (Mehrotra and Ravichandran 2022), were strengthened in aged retina. This may be due to increased Gas6 expression, thereby enhancing interactions between microglia and other retinal cells. In support of the sc‐RNA‐seq findings, Gas6 protein expression level in aged retina was significantly higher, whereas Axl and MerTK expression were lower than those in young retina. We speculated that this may be due to aged retina having increased apoptosis, along with decreased microglial efferocytosis, resulting in a positive feedback loop to further stimulate apoptotic cell Gas6 expression. As for microglial MerTK and Axl, they are mainly involved in apoptotic cell recognition (Burstyn‐Cohen and Fresia 2023; Doran et al. 2020), and numerous studies have shown that decreased MerTK and Axl expression could impair efferocytosis (Gao et al. 2023; Justynski et al. 2023); for instance, Hu et al. showed that decreased MerTK in aged versus young macrophages, in turn, reduces aged macrophages ability to recognize apoptotic cells (Hu et al. 2023). Decreased MerTK and Axl expression, along with impaired efferocytosis in aging, were also found in our study.

To verify our sc‐RNA‐seq findings, efferocytosis was compared between young and aged microglia, both in vitro and in vivo. For the in vitro experiments, DOX, an anti‐cancer drug that could induce microglial senescence (Wei et al. 2023), was used, and DOX‐treated BV2 microglia had significantly lowered efferocytosis. Likewise, the in vivo model involved I/R injury, which results in large numbers of apoptotic cells being generated in a short time; these cells would normally be removed by effective microglial efferocytosis. Our results revealed that Gas6^+^TUNEL^+^ cells were significantly elevated in aged retina after I/R injury, which may be due to aged retinas being less resistant to the insult, leading to increased apoptosis. However, our findings of MerTK and Axl being downregulated in aged microglia indicated that impaired efferocytosis may also contribute to increased Gas6^+^TUNEL^+^. Furthermore, by quantifying the co‐localization of Iba‐1^+^ microglia with TUNEL^+^ apoptotic retinal cells, we found that aged microglia had impaired efferocytosis of apoptotic cells post‐I/R, supporting the sc‐RNA‐seq analysis. This was in line with Aprahamian et al. who reported that aging was related to impairments in removing apoptotic keratinocytes (Aprahamian et al. 2008), as well as Thomas et al. who showed that naturally aged microglia had decreased amyloid‐β phagocytic capacity (Thomas et al. 2022). Additionally, De Maeyer et al. found that efferocytosis of apoptotic cells by macrophages was reduced with aging, impairing inflammatory resolution among elderly individuals (De Maeyer et al. 2020). These studies therefore supported our observations that aging impaired microglial efferocytosis of apoptotic retinal cells. In addition, the age‐related increase in retinal microglia was confirmed by the results of retinal flatmounts. However, this increase in microglial number was not associated with enhanced efferocytosis. In aged microglia, the downregulation of receptors such as MerTK and Axl resulted in reduced clearance of apoptotic cells.

To elucidate the complex molecular mechanisms involved in microglial efferocytosis, we applied sc‐RNA‐seq to analyze DEGs between young and aged microglia in the efferocytosis‐related subcluster. We found that a large number of genes previously documented to be associated with efferocytosis (Zhang et al. 2019, 2022; Liao et al. 2023; Xu et al. 2024; Chen et al. 2024), including Myl12a, Atp6v1b2, Dock10, Vav1, Creb1, Rac2, Msr1, and Mapk1, were downregulated in aged microglia. Additionally, an in vitro study showed that efferocytosis stimulates phagocytes to produce transforming growth factor‐β (TGFβ) (Blander 2017), which was in line with our findings demonstrating that the genes downregulated in aged microglia Subcluster 4 were related to TGFβ signaling, which might be associated with defective efferocytosis. With respect to specific mechanisms behind lowered efferocytosis, a number of possible ones have been described in the literature, such as by Poon et al. who noted that aging was associated with lowered clearance capabilities of phagocytes, resulting in increased apoptotic retinal cell accumulation (Poon and Ravichandran 2024). This may be due to increased MerTK cleavage in aged versus young phagocytes, in turn reducing aged phagocyte ability to recognize apoptotic cells (Rymut et al. 2020). Indeed, our in vitro model found that even though aged microglia have stronger MerTK and Gas6 interactions, compared to young ones, MerTK expression was significantly lower in BV2 + DOX, suggesting that decreases in functional MerTK lowered the availability of MerTK to interact with Gas6 for triggering efferocytosis. Furthermore, efferocyte binding to apoptotic cells has been noted in a number of studies to be able to suppress TNF and IL‐6 production, via inhibiting NF‐κB activation (Yang et al. 2015; Doran et al. 2020). This is in line with our observations, in which both NF‐κB and inflammatory‐related signaling pathways were upregulated in aged microglia. Based on these findings, we postulated that MerTK downregulation reduced IKK suppression, thereby leading to increased NF‐κB activity and dependent inflammatory cytokine production. However, the precise association between lowered MerTK activity with lowered efferocytosis, along with increased inflammatory cytokine and apoptotic cell accumulation, as well as the pathways involved, in aged retinas, should be further elucidated in future studies. Nevertheless, microglial efferocytosis plays an important role in promoting inflammatory resolution and maintaining retina homeostasis (Doran et al. 2020). Therefore, therapeutic strategies designed to improve efferocytosis might restrain the development and progression of retinal diseases.

We also observed, consistent with prior sc‐RNA‐seq analyses of freshly dissociated retinal tissues, that rod photoreceptors are the predominant cell population, with microglia and RGCs in small proportions, which aligns with the expected retinal cell composition (Peng et al. 2019; Wang et al. 2024). However, for sampling cell types with low proportions, cell–cell variability is unavoidable. Consequently, to reduce the impact of this limitation, we implemented stringent cut‐offs of *p* < 0.01, |log_2_FC| ≥ 0.26, and gene detected in > 10% of cells within a specific cluster, to minimize false positives when identifying DEGs. Nevertheless, future studies should increase microglial cell numbers, remove rod photoreceptors from the retinal cell suspension (Peng et al. 2019), in order to fully examine low‐abundance cell types. Another limitation is that even though we found that the proportion of rod cells decreased in aged retina, suggesting potential photoreceptor degeneration, this was not examined in detail. Therefore, future studies should be conducted to investigate the relationship between microglial efferocytosis and photoreceptor degeneration.

## 5 Conclusions

In this study, sc‐RNA‐seq was applied to identify different retinal cell types and their proportions among young and aged mice. We found that in aged retinas, the proportions of retinal cells changed, particularly for microglia and RGCs. Additionally, large numbers of DEGs were found between young and aged retinas for amacrine, horizontal, Müller, astrocytes, microglia, VEC and RGCs; these DEGs were enriched for cellular senescence and apoptosis. Furthermore, compared to young, aged microglia had increased numbers of interactions with other retinal cells, and an efferocytosis‐associated microglia subcluster was successfully identified based on specific gene expression. Lastly, aged microglia had lowered efferocytosis, which was found at the single‐cell level and further verified in vitro among DOX‐induced senescent BV2, as well as in vivo in a retinal I/R injury mouse model.

## Author Contributions

P.L., Q.W., H.Y., and Z.S. conceived and designed the research. S.W., Y.L., Q.C., W.Q., X.L., X.Y., and Y.J. performed the experiments. P.L., Q.W., and S.W. analyzed the data. P.L. interpreted the data and wrote the manuscript. Q.W. and Z.S. revised the manuscript. All authors have read and approved the final manuscript.

## Conflicts of Interest

The authors declare no conflicts of interest.

## Supporting information


Appendix S1

## Data Availability

The datasets used during this study are available from the corresponding author upon reasonable request.
