# Supplementary material for: Single‐Cell RNA‐Seq Reveals Aging‐Related Impairment of Microglial Efferocytosis Contributing to Apoptotic Cells Accumulation After Retinal Injury
Source: Aging Cell. 2025 May 15;24(8):e70097. doi: 10.1111/acel.70097 (PMC12341814; doi:10.1111/acel.70097)
Supplement: Supplementary file 1 — Appendix S1 [file ACEL-24-e70097-s001.docx]

**Supplementary File**

for

**Single‐cell RNA‐seq reveals aging-related impairment of microglial efferocytosis contributing to apoptotic cells accumulation after retinal injury**

Pan Liu^1,2†^, Qi Wang^1,2†^, Shuimiao Wang^1,2^, Ying Liu^1,2^, Qiqi Chen^1,2^, Wanyun Qin^1^, Xinna Liu^1^, Xinqi Ye^1,2^, Yexuan Jiao^1^, Huiping Yuan^1*^, and Zhengbo Shao^1,2*^

^1^Department of Ophthalmology, The Second Affiliated Hospital of Harbin Medical University, Harbin, China.

^2^Future Medical Laboratory, The Second Affiliated Hospital of Harbin Medical University, Harbin, China.

^†^Pan Liu and Qi Wang have equally contributed to this work.

Zhengbo Shao is the lead contact.

^*^Correspondence:

Zhengbo Shao, Department of Ophthalmology, The Second Affiliated Hospital of Harbin Medical University, Harbin, China.

Email: shaozhengbohmu@126.com

Huiping Yuan, Department of Ophthalmology, The Second Affiliated Hospital of Harbin Medical University, Harbin, China.

Email: yuanhp2013@126.com

**Supplementary Table**

**TABLE S1** Numbers of cells sequenced among 35 distinct clusters in young and aged retinas.

| Cluster | Young1 | Young2 | Young3 | Aged1 | Aged2 | Aged3 |
| --- | --- | --- | --- | --- | --- | --- |
| 0 | 760 | 837 | 1003 | 1178 | 1372 | 725 |
| 1 | 1295 | 654 | 1196 | 981 | 856 | 885 |
| 2 | 671 | 930 | 881 | 1314 | 775 | 840 |
| 3 | 672 | 1036 | 1020 | 677 | 865 | 726 |
| 4 | 481 | 984 | 638 | 604 | 857 | 506 |
| 5 | 732 | 911 | 740 | 687 | 490 | 629 |
| 6 | 799 | 838 | 848 | 668 | 503 | 618 |
| 7 | 630 | 1771 | 949 | 304 | 493 | 369 |
| 8 | 612 | 698 | 717 | 566 | 603 | 488 |
| 9 | 436 | 252 | 426 | 678 | 470 | 335 |
| 10 | 378 | 1109 | 557 | 411 | 253 | 432 |
| 11 | 544 | 375 | 465 | 287 | 830 | 281 |
| 12 | 370 | 372 | 465 | 369 | 471 | 341 |
| 13 | 319 | 337 | 305 | 432 | 405 | 134 |
| 14 | 405 | 255 | 499 | 361 | 338 | 583 |
| 15 | 174 | 229 | 138 | 432 | 317 | 269 |
| 16 | 244 | 265 | 275 | 375 | 327 | 195 |
| 17 | 205 | 222 | 205 | 500 | 239 | 513 |
| 18 | 319 | 560 | 523 | 109 | 142 | 141 |
| 19 | 153 | 197 | 121 | 410 | 245 | 174 |
| 20 | 235 | 255 | 183 | 263 | 180 | 178 |
| 21 | 190 | 201 | 143 | 201 | 280 | 184 |
| 22 | 131 | 173 | 85 | 194 | 152 | 105 |
| 23 | 106 | 134 | 72 | 167 | 150 | 90 |
| 24 | 108 | 136 | 78 | 188 | 156 | 100 |
| 25 | 72 | 99 | 61 | 162 | 109 | 65 |
| 26 | 74 | 50 | 22 | 277 | 131 | 63 |
| 27 | 83 | 101 | 46 | 103 | 74 | 16 |
| 28 | 38 | 85 | 93 | 119 | 76 | 35 |
| 29 | 38 | 64 | 27 | 79 | 42 | 64 |
| 30 | 28 | 36 | 27 | 87 | 37 | 44 |
| 31 | 63 | 36 | 29 | 47 | 29 | 15 |
| 32 | 30 | 35 | 22 | 26 | 13 | 26 |
| 33 | 38 | 15 | 24 | 10 | 14 | 4 |
| 34 | 16 | 6 | 22 | 10 | 16 | 1 |
| 35 | 11 | 1 | 1 | 7 | 11 | 9 |

Supplementary Figures

**
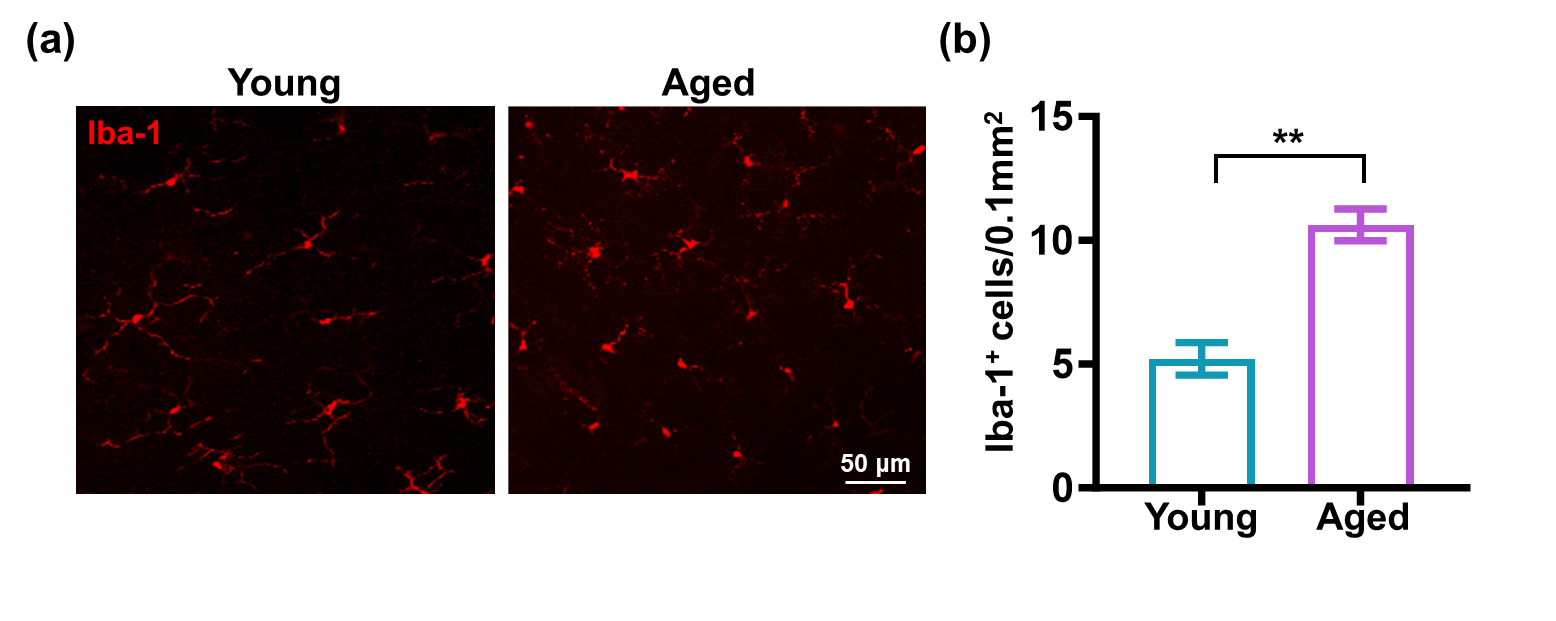
**

**FIGURE S1** Microglial numbers between young and aged retinas. (a) Representative confocal images of retinal mounts and (b) quantification of ionized calcium-binding adapter molecule (Iba)-1^+^ cells in young and aged retinas. n=3/group. ***p*<0.01.

**
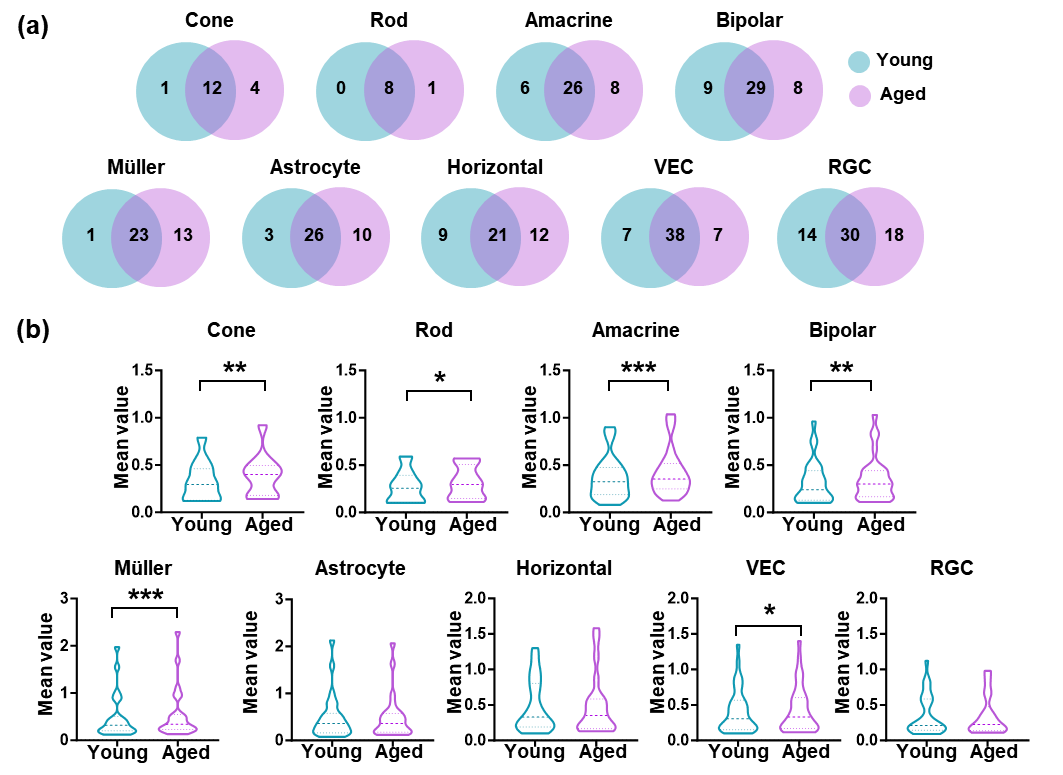
**

**FIGURE S2** Analyses of interactions between microglia and other retinal cell types in young and aged retinas. (a) Venn diagrams illustrating the number of cell-cell interactions between microglia and other cell types, including those specific to either young or aged retinas, as well as ones shared by both. (b) Violin plots showing the strength of interactions between microglial with other cell types in young and aged retinas. n=8-38/group. **p*<0.05, ***p*<0.01, ****p*<0.001.

**
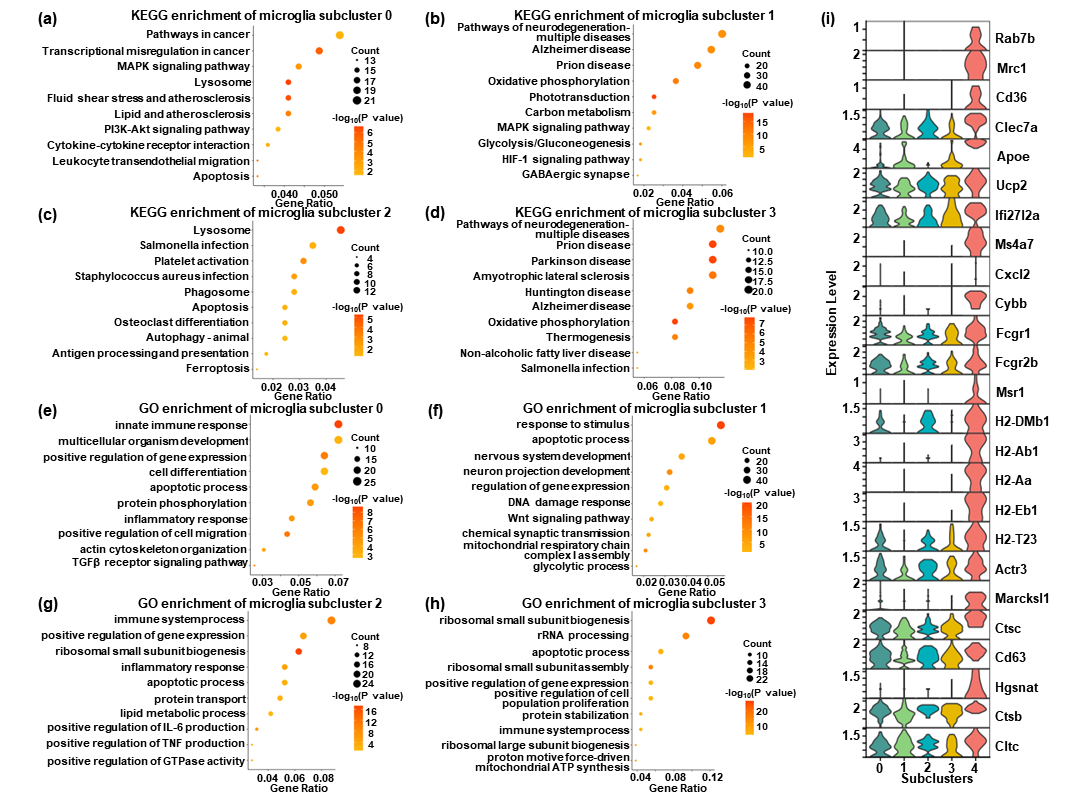
**

**FIGURE S3** Analyses of differentially expressed genes (DEGs) for microglial subclusters 0-3 identified by partial cell clustering of 259 young and aged microglia, as well as efferocytosis-related gene expression. Kyoto Genes and Genomes (KEGG) enrichment analyses of DEGs from microglial subclusters (a) 0, (b) 1, (c) 2, and (d) 3. Gene ontology (GO) enrichment analyses of DEGs from microglial subclusters (e) 0, (f) 1, (g) 2, and (h) 3. (i) Violin plots of the expression levels for efferocytosis-related genes among microglial subclusters 0-4. n=3/group.


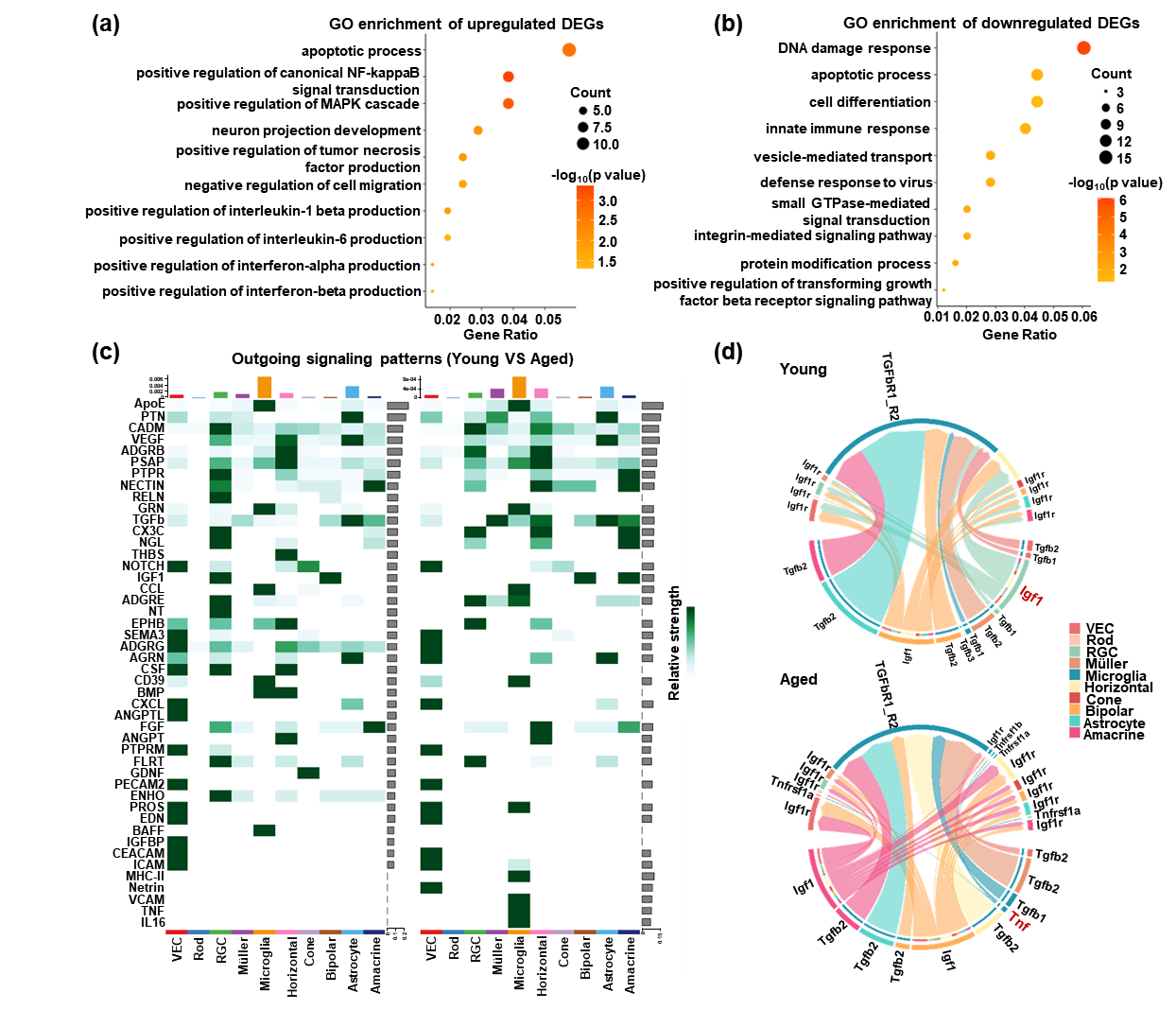


**FIGURE S4** Bubble plots of GO enrichment analyses for (a) up- and (b) down-regulated DEGs among young versus aged microglial subcluster 4. (c) Heatmaps depicting outgoing signaling patterns for different retinal cell types with microglial subcluster 4 in young versus aged retinas. (d) Ligand-receptor interactions between microglial subcluster 4 and other retinal cell types in young versus aged retinas. n=3/group.

**
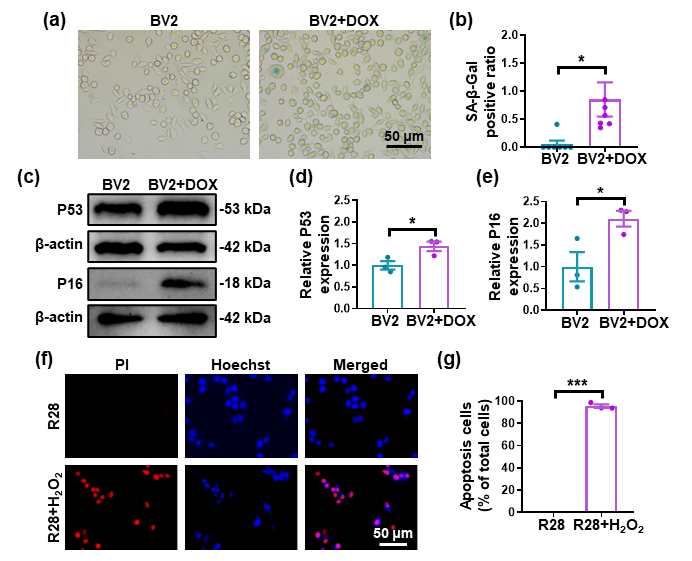
**

**FIGURE S5** Identification of successful induction of senescence for BV2 murine microglia and apoptosis for R28 rat retinal cells *in vitro*. (a) Senescence-associated beta-galactosidase (SA-β-gal) staining images, as well as (b) quantification of SA-β-gal^+^ BV2 cells, between untreated control (BV2), and senescence inducer doxorubicin (DOX)-treated BV2 (BV2+DOX) groups. (c) Western blot image, as well as quantification of senescence markers (d) p53 and (e) p16 protein expression between the 2 groups. (f) Representative immunofluorescence images of Hoechst/propidium iodide (PI), as well as (g) quantification of apoptotic PI^+^ R28 retinal cells, as a percentage of total cells, between untreated (R28) and H_2_O_2_-treated (R28+H_2_O_2_) R28 groups. Protein expression was normalized to β-actin. Data are expressed as mean±standard deviation (SEM). n=3/group. **p* < 0.05, ****p* < 0.001.


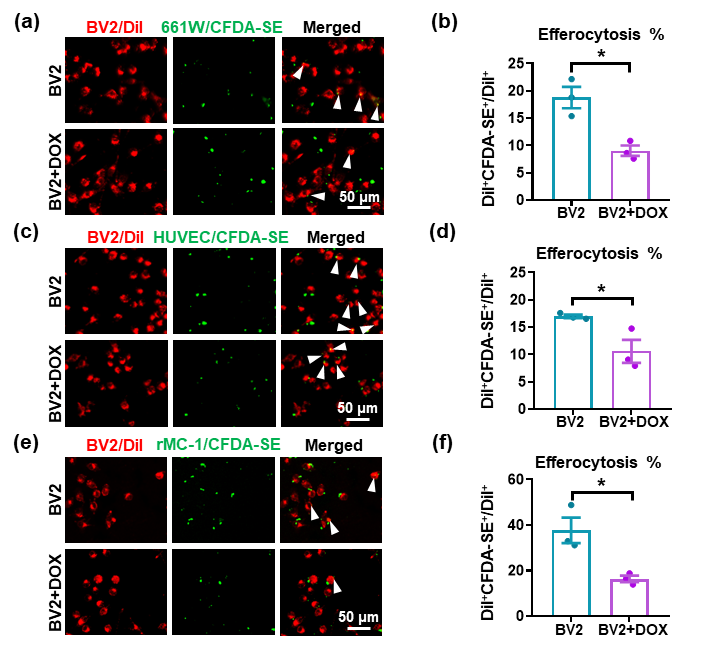


**FIGURE S6** (a) Representative immunofluorescence staining images and (b) quantification of Dil^+^carboxyfluorescein diacetate (CFDA)^+^ cells, representing successful efferocytosis of H_2_O_2_-treated apoptotic CFDA^+^ 661W (retinal photoreceptor cell line) by Dil^+^BV2 microglia, as a percentage of total Dil^+^, between untreated control (BV2) and DOX-treated senescent BV2 (BV2+DOX) groups. (c) Representative immunofluorescence staining images and (d) Dil^+^CFDA^+^ quantification, representing successful efferocytosis of H_2_O_2_-treated apoptotic CFDA^+^ human umbilical cord vein endothelial cells (HUVECs) by Dil^+^BV2 microglia, as a percentage of total Dil^+^, between BV2 and BV2+DOX. (e) Representative immunofluorescence staining images and (f) Dil^+^CFDA^+^ quantification, representing successful efferocytosis of H_2_O_2_-treated apoptotic CFDA^+^ rMC-1 (rat retinal Müller) by Dil^+^BV2 microglia, as a percentage of total Dil^+^, between BV2 and BV2+DOX. n=3/group. **p* < 0.05.


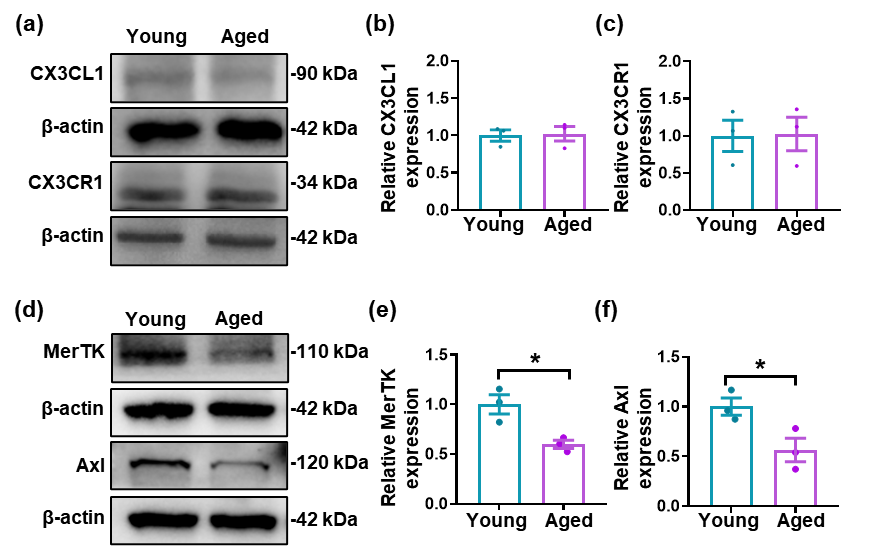


**FIGURE S7** Efferocytosis-related receptor and ligand expression, under normal physiological conditions, between young and old retinas. (a) Representative Western blot images and quantification of (b) CX3CL1 and (c) CX3CR1 protein expression levels between the 2 groups. (d) Representative Western blot images and quantification of (e) MerTK and (f) Axl protein expression levels between the 2 groups. n=3/group. **p*<0.05.

**
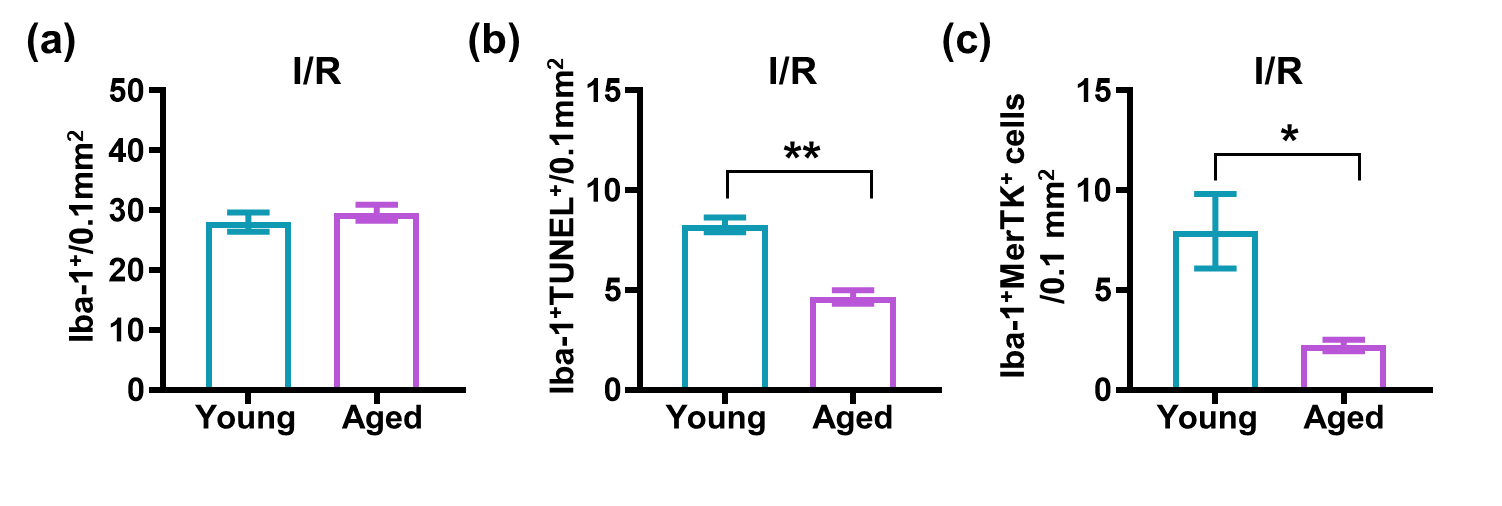
**

**FIGURE S8** Quantification of (a) Iba-1^+^ microglia, (b) Iba-1^+^TUNEL^+^, and (c) Iba-1^+^MerTK^+^ cells, between young and aged retinas, on day 3 post-I/R injury. n=3/group. **p*<0.05, ***p*<0.01.
